# Binary temporal upconversion codes of Mn^2+^-activated nanoparticles for multilevel anti-counterfeiting

**DOI:** 10.1038/s41467-017-00916-7

**Published:** 2017-10-12

**Authors:** Xiaowang Liu, Yu Wang, Xiyan Li, Zhigao Yi, Renren Deng, Liangliang Liang, Xiaoji Xie, Daniel T. B. Loong, Shuyan Song, Dianyuan Fan, Angelo H. All, Hongjie Zhang, Ling Huang, Xiaogang Liu

**Affiliations:** 1 Department of Chemistry, National University of Singapore, Singapore, 117543 Singapore; 2 SZU-NUS Collaborative Innovation Center for Optoelectronic Science & Technology, Key Laboratory of Optoelectronic Devices and Systems of Ministry of Education and Guangdong Province, College of Optoelectronic Engineering, Shenzhen University, Shenzhen, 518060 China; 3 Key Laboratory of Flexible Electronics & Institute of Advanced Materials, Jiangsu National Synergetic Innovation Center for Advanced Materials, Nanjing Tech University, Nanjing, 211816 China; 4 Singapore Institute of Neurotechnology (SINAPSE), National University of Singapore, Singapore, 117456 Singapore; 5 State Key Laboratory of Rare Earth Resource Utilization, Changchun Institute of Applied Chemistry, Chinese Academy of Sciences, Changchun, 130022 China; 6 Department of Biomedical Engineering, Johns Hopkins School of Medicine, Baltimore, MD 21205 USA; 7 Department of Neurology, Johns Hopkins School of Medicine, Baltimore, MD 21205 USA; 8 Institute of Materials Research and Engineering, Agency for Science, Technology and Research, Singapore, 117602 Singapore

## Abstract

Optical characteristics of luminescent materials, such as emission profile and lifetime, play an important role in their applications in optical data storage, document security, diagnostics, and therapeutics. Lanthanide-doped upconversion nanoparticles are particularly suitable for such applications due to their inherent optical properties, including large anti-Stokes shift, distinguishable spectroscopic fingerprint, and long luminescence lifetime. However, conventional upconversion nanoparticles have a limited capacity for information storage or complexity to prevent counterfeiting. Here, we demonstrate that integration of long-lived Mn^2+^ upconversion emission and relatively short-lived lanthanide upconversion emission in a particulate platform allows the generation of binary temporal codes for efficient data encoding. Precise control of the particle’s structure allows the excitation feasible both under 980 and 808 nm irradiation. We find that the as-prepared Mn^2+^-doped nanoparticles are especially useful for multilevel anti-counterfeiting with high-throughput rate of authentication and without the need for complex time-gated decoding instrumentation.

## Abstract (web summary)

Luminescent materials that are capable of binary temporal coding are desirable for multilevel anti-counterfeiting. Here, the authors engineer nanoparticles that produce binary color codes on different timescales by combining the long-lived luminescence of Mn^2+^ with the relatively short-lived emission of lanthanides.

## Introduction

The emission color of luminescent materials plays a crucial role in encoding information for anti-counterfeiting^1–3^ and optical multiplexing^4, 5^. Lanthanide-doped upconversion nanoparticles are particularly suitable for such applications because they can be readily deposited or patterned as films from solution to create multicolor barcodes under a single-wavelength excitation or through thermal radiation^6–10^. For example, three primary colors can be readily obtained through the use of Er-Tm (red), Yb-Er (green), and Yb-Tm (blue) dopant pairs^11–13^. In addition, the strong dependence of the color output on the excitation source, such as power density, pulse duration and excitation wavelength, provides an added benefit in fine-tuning the emission profiles without the need for particle composition modification^14–18^. Furthermore, a low threshold of pumping (down to 1 W cm^−2^) is required to realize upconversion emission, making these nanoparticles an ideal target for practical and high-capacity information storage^19–22^.

Despite the attractions, the use of color elements for multiplexing to enhance data storage density and security remains a formidable challenge. An obvious bottleneck is the unavoidable overlap in the emission spectra of the nanoparticles under study. Alternatively, time-domain codes of lanthanide-doped nanoparticles have proven effective in adding flexibility in high-density data storage and another dimension of complexity to combat counterfeiting^23–25^. However, the need for time-gated instrumentation and a tedious data-decoding process poses a considerable constraint for practical use. One promising strategy that simplifies the coding and decoding procedure is the integration of long-lived emission (>15 ms) with conventional upconversion emission for distinct binary temporal scales that can be visualized on excitation at a single wavelength.

Optical nanoparticles that simultaneously display a long-lived emission and a relatively short-lived emission are generally difficult to prepare by direct coating of conventional afterglow materials such as MAl_2_O_4_:Eu^2+^/Dy^3+^ (M=Ca or Sr) onto lanthanide-doped nanocrystals, typically composed of NaGd(or Y)F_4_:Yb/Er(or Tm). This difficulty is largely due to the challenge of mitigating the large lattice mismatch between the two materials. Co-doping of transition metal ions having a long-lived emission nature and lanthanide dopants within a host lattice has also proven ineffective owing to the weak energy transfer between the two optical centers^26^. In addition, optical incompatibility between the pair in some cases can even lead to deleterious cross-relaxation, resulting in rapid quenching of excitation energies^27–29^.

Here, we reason that upconversion nanoparticles possessing binary color scales can be obtained by spatially controlled co-doping of manganese (II) ions into hexagonal-phased NaLnF_4_ (Ln = lanthanide) lattices. First, the spin-forbidden transition nature of the 3*d* ^5^ configuration of Mn^2+^ permits long-lived emission up to several milliseconds, a duration which is discernible by the naked eye^30–33^. Furthermore, the low phonon energy of NaLnF_4_ lattice is likely to decrease the probability of nonradiative transitions, thereby leading to enhanced upconversion efficiency for both emitters. By making use of energy migrators to link the two emitting centers, we report the access to binary temporal upconversion codes benefiting from the large lifetime difference in their emissions. We find that a precise control over core-shell structures not only allows for the creation of a library of short-lived color codes but also makes it possible to excite the nanoparticles under both 980 and 808 nm irradiation^34–42^. We show that these nanomaterials are particularly attractive for multilevel anti-counterfeiting by taking advantage of the parallel implementation of optical readout at high-throughput rates.

## Results

### Synthesis of basic binary temporal upconversion codes

**Fig. 1:**
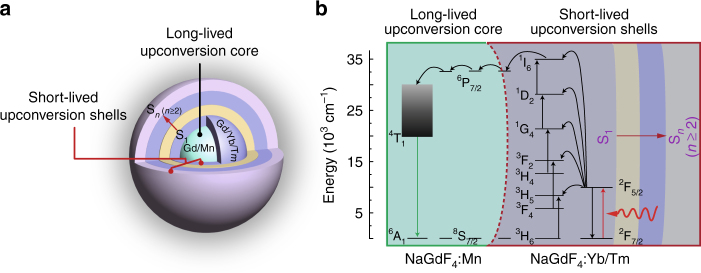
Rational design of binary temporal upconversion codes through Mn^2+^ codoping. **a** Structure design of a multilayer nanoparticle for simultaneously displaying short- and long-lived upconversion emissions. Noted that NaGdF_4_:Mn(30 mol%) and NaGdF_4_:Yb/Tm(49/1 mol%) are exploited as core and first shell (S_1_) of the multilayer nanoparticle to achieve long-lived Mn^2+^ luminescence through Gd-sublattice-mediated energy migration. Other characteristic lanthanide emissions from the nanoparticle can be realized by further coating additional shell layers (S_*n*_) with different lanthanide compositions: strategy i S_2_ = NaYF_4_, ii S_2_ = NaYF_4_:A (A = Eu^3+^, Eu^3+^/Tb^3+^ or Tb^3+^), iii S_2_ = NaYF_4_, S_3_ = NaYF_4_:Yb/Er (5/0.05, 20/2 or 50/0.05 mol%), iv S_2_ = NaYF_4_:Nd (20 mol%), S_3_ = NaYF_4_:Nd/Yb/Er(1/30/0.5 or 2/10/1 mol%), and v S_2_ = NaYF_4_, S_3_ = NaYF_4_:Nd (20 mol%), S_4_ = NaYF_4_:Nd/Yb/Er(2/10/1 mol%). **b** Proposed energy transfer pathway between the core and shell layers accounting for the simultaneous generation of short- and long-lived upconversion luminescence under excitation at 980 nm

The particle design for achieving long-lived upconversion encoding is shown in Fig. 1a. Hexagonal-phased NaGdF_4_:Mn and NaGdF_4_:Yb/Tm(49/1 mol%) are used as core and first shell layer, respectively, to enable Mn^2+^ emission through an energy migration upconversion process (Fig. 1b)^43–45^. Succeeding layers made of pure or doped NaYF_4_ materials can be conveniently passivated in support of color tuning of the short-lived lanthanide emission while preserving the long-lived luminescence of Mn^2+^.

To validate our hypothesis, we first prepared hexagonal-phased NaGdF_4_:Mn core nanoparticles by a hydrothermal method. In the synthesis, we observed a hexagonal-to-cubic phase transformation at a high doping concentration of Mn^2+^ (40 mol%)^27, 29^, as revealed by powder X-ray diffraction studies (Supplementary Fig. 1). Inductively coupled plasma atomic emission spectroscopy analysis showed that there are big discrepancies between the designed and measured concentrations of Mn^2+^ in core nanoparticles (Supplementary Fig. 2 and Supplementary Table 1), suggesting the low solubility of Mn^2+^ in the hexagonal-phased NaGdF_4_.

**Fig. 2:**
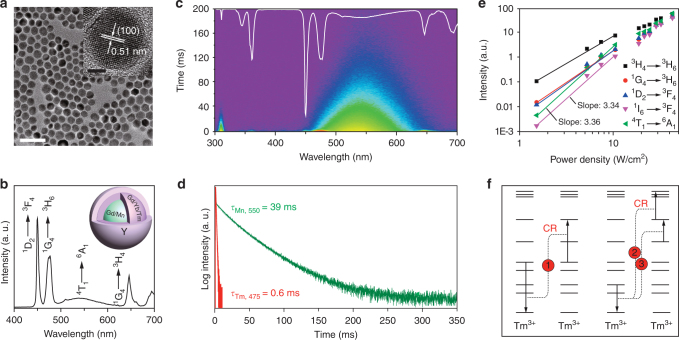
Characterization of multilayer nanoparticles of NaGdF_4_:Mn (30 mol%)@NaGdF_4_:Yb/Tm (49/1 mol%)@NaYF_4_. **a** TEM image of the as-prepared upconversion nanoparticles, *scale bar*, 50 nm. (*Inset*: high-resolution TEM imaging of a single core-shell-shell nanoparticle, *scale bar*, 5 nm). **b** Emission profile of the as-prepared nanoparticles under excitation at 980 nm (power density: 30 W cm^−2^). **c** Time-resolved spectrum of the as-prepared Mn^2+^-doped upconversion nanoparticles. **d** Lifetime comparison of Mn^2+^ emission (550 nm, ^4^T_1_ → ^6^A_1_) and Tm^3+^ (475 nm, ^1^D_2_ → ^3^F_4_) of the as-prepared Mn^2+^-doped nanoparticles recorded in aqueous solution at room temperature. **e** Power density dependence of the upconverted Tm^3+^ and Mn^2+^ emissions. Note that the slopes of power-dependent emission centered at 345 nm for Tm^3+^ and at 550 nm for Mn^2+^ are measured to be 3.34 and 3.36, respectively. **f** Proposed cross-relaxation (CR) processes between two neighboring Tm^3+^ ions, accounting for the experimentally observed lower exponential power dependence of the Tm^3+^ and Mn^2+^ emissions

We next performed epitaxial growth of NaYF_4_:Yb/Tm(49/1 mol%) and NaYF_4_ layers onto the as-prepared NaGdF_4_:Mn(30 mol%) cores by a combination of coprecipitation and thermal decomposition methods (Supplementary Fig. 3). After implementing the epitaxial growth, the resultant nanoparticles were confirmed to retain hexagonal phase (Supplementary Fig. 1). Transmission electron microscopy (TEM) showed that the epitaxial growth led to an obvious increase in the size of the nanoparticles from 12 to 17 nm (Fig. 2a), accompanying with a slight morphological change from short-rod-like to spherical shape. The morphological change can be ascribed to the occurrence of oleic acid-assisted etching during the high-temperature shell-growth process (290 °C)^46^. High-resolution TEM imaging revealed the single-crystalline nature of the as-prepared core-shell-shell nanoparticles with a measured *d*-spacing of 0.51 nm (Fig. 2a, inset), which is in good agreement with the lattice spacing in the (100) planes of hexagonal phase NaGdF_4_.

Optical characterization showed a gradual increase in the emission intensity upon subsequent growth of NaYF_4_:Yb/Tm(49/1 mol%) and NaYF_4_ layers onto the as-prepared NaGdF_4_:Mn(30 mol%) core nanoparticles (Supplementary Fig. 4), implying the formation of core-shell and core-shell-shell nanoparticles. The emission profile of the resulting multilayer nanoparticles (Fig. 2b) showed a broad Mn^2+^ emission in the region of 490–625 nm, in addition to characteristic emission bands for Tm^3+^ centered at 450, 475, 646, and 694 nm. The generation of upconversion Mn^2+^ luminescence is likely a result of Gd-sublattice-mediated energy migration, made evident by the observation of a notable decrease in the lifetime of Gd^3+^ (311 nm, ^6^P_7/2_ → ^8^S_7/2_) from 6.5 to 4 ms after the placement of Mn^2+^ ions into the particle’s core (Supplementary Fig. 5). It was found that the upconversion emission intensity of Mn^2+^ is highly dependent on the doping level (2.5–30 mol%) of Mn^2+^ in the core (Supplementary Fig. 6). On a separate note, the emission intensity of Mn^2+^ ions under investigation is about ten times stronger than that obtained from cubic-phased equivalents (Supplementary Figs 7 and 8). Taken together, these results suggest that hexagonal-phased NaGdF_4_ lattice is more suitable for achieving energy transfer and energy migration upconversion for both lanthanides and transition metal ions, as opposed to cubic-phased counterpart.

The lifetime of Mn^2+^ emission was found to be much longer than that of Tm^3+^ emission (Fig. 2c). As expected, we did observe a strong green luminescence after disappearance of the short-lived Tm^3+^ emission. The lifetime of Mn^2+^ emission from the multilayer nanoparticles was estimated to be 39 ms, which is about 65 times longer than that of Tm^3+^ emission centered at 475 nm (Fig. 2d). We ascribed the long-lived Mn^2+^ upconversion emission mainly to the spin-forbidden nature of its ^4^T_1_ → ^6^A_1_ transition. In addition, the multilayer structure of the nanoparticle should also partially contribute to the long lifetime of the Mn^2+^ upconversion emission because this design can effectively lead to the separation of Yb^3+^ and Mn^2+^ and thereby prevent the back-energy transfer from Mn^2+^ to Yb^3+^ (ref. ^47^). The prevention of the Mn^2+^-to-Yb^3+^ back-energy transfer is likely to be crucial for retaining the intrinsically long-lifetime of Mn^2+^ upconversion emission. For example, a much shorter lifetime of Mn^2+^ upconversion emission (19 ms) was observed in homogeneously doped nanoparticles of NaYF_4_:Yb/Mn(5/30 mol%) (Supplementary Fig. 9).

### Simulation studies

To provide insight into the energy transfer within the Mn^2+^-doped nanoparticles, we performed power-dependent study of the upconverted Tm^3+^ and Mn^2+^ emissions. We recorded a slope of 3.34 for the violet emission of Tm^3+^ centered at 345 nm and a slope of 3.36 for the green emission of Mn^2+^ centered at 550 nm (Fig. 2e). This result is quite surprising because we expect that a five-photon excitation should be the dominant process as outlined in Fig. 1b. To clarify this point, we further carried out energy transfer simulation, with or without consideration of Tm^3+^-Tm^3+^ cross-relaxation, by employing a set of rate equations (Supplementary Figs. 10 and 11). Interestingly, the simulated results with the consideration of the Tm^3+^-Tm^3+^ cross-relaxation are in good agreement with our experimental results (Fig. 2f), as supported by the generation of similar slopes in the fitting curves within the low power density region. Taken together, these findings suggest that the green upconversion emission of Mn^2+^ is likely to be the result of a five-photon upconversion process; and the experimentally observed lower exponential power dependence of the emission is due to the occurrence of cross-relaxation between neighboring Tm^3+^ ions (Supplementary Note 1).

### Enriching the variety of short-lived upconversion codes

**Fig. 3:**
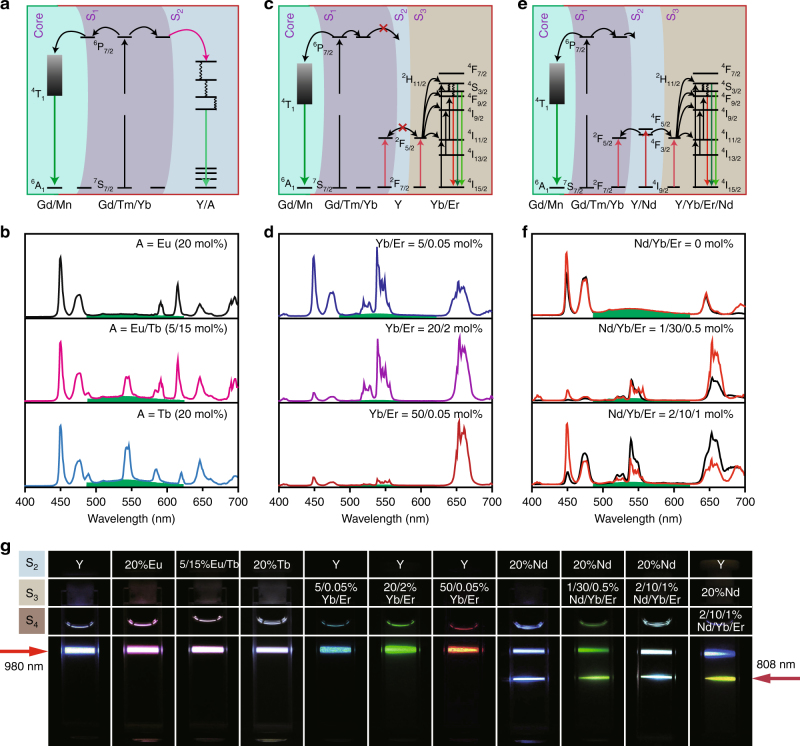
Synthetic strategies for tuning short-lived upconversion emission. **a** Doping of lanthanide activators (A = Eu^3+^, Eu^3+^/Tb^3+^ or Tb^3+^) into the outmost shell (S_2_) of NaYF_4_ to realize interfacial energy transfer. In contrast to energy migration strategy, interfacial energy transfer mainly occurs at the interface and has less effect on the Mn^2+^ luminescence. **b** Representative emission profiles of the as-prepared NaGdF_4_:Mn(30 mol%)@NaGdF_4_:Yb/Tm(49/1 mol%)@NaYF_4_:A (A = Eu^3+^, Eu^3+^/Tb^3+^ or Tb^3+^) nanoparticles. **c** Strategy involving the addition of NaYF_4_ (S_2_) and NaYF_4_:Yb/Er (S_3_) layers. Note that the emission dependence of the NaYF_4_:Yb/Er shell on the relative doping level of Yb/Er provides an additional means to modulate the emission color of the nanoparticles. **d** Emission profiles of the as-prepared NaGdF_4_:Mn(30 mol%)@NaGdF_4_:Yb/Tm(49/1 mol%)@NaYF_4_@NaYF_4_:Yb/Er(5/0.05, 20/2 or 50/0.05) nanoparticles. **e** Doping of Nd^3+^ into the NaYF_4_ shell (S_2_) allows the excitation to be carried out either under 980 or 808 nm. Notably, the small-sized and curved arrow between S_1_ and S_2_ layers is used to represent a much weaker interfacial energy transfer from Gd^3+^ to Nd^3+^ relative to energy migration from Gd^3+^ in the S_1_ layer to Mn^2+^ in the core. **f** Emission spectra of NaGdF_4_:Mn(30 mol%)@NaGdF_4_:Yb/Tm(49/1 mol%)@NaYF_4_:Nd(20 mol%) nanoparticles and the corresponding multilayer nanoparticles passivated with NaYF_4_:Nd/Yb/Er (*x* mol%) (*x* = 1/30/0.5 or 2/10/1) under excitation at 980 (*red curve*) and 808 nm (*black curve*). The pump powers of the 980 and 808 nm lasers were fixed at 1 and 4 W for spectral measurement, respectively. **g** Luminescence photographs showing multicolour tuning of the steady upconversion of the as-prepared nanoparticles under excitation at 980 or 808 nm. The energy transfer from Nd^3+^  → Yb^3+^  → Tm^3+^  → Gd^3+^  → Mn^2+^ under excitation at 808 nm can be largely suppressed by growth of an inert shell between NaYF_4_:Nd (20 mol%) and NaGdF_4_:Yb/Tm(49/1 mol%), and thus different color outputs can be generated upon excitation at 980 and 808 nm (Supplementary Fig. 19)

To add more flexibility into tuning the short-lived upconversion emission of the Mn^2+^-doped nanoparticles, we set out to examine the possibility of doping lanthanide activators (denoted as A: Eu^3+^, Eu^3+^/Tb^3+^, or Tb^3+^) into the outmost shell of NaYF_4_ (Fig. 3a and Supplementary Fig. 12). As anticipated, an interfacial energy transfer from Gd^3+^ to a given lanthanide activator occurred, leading to a characteristic emission band of the activator in the visible region (Fig. 3b). Importantly, the variation in composition had a marginal influence on the optical properties of Mn^2+^ ions (Supplementary Fig. 13). In contrast, an energy migration transfer process involving doping of Tb^3+^ (20 mol%) into the outmost layer of NaGdF_4_ led to a substantial decrease in the emission lifetime (Supplementary Fig. 14). The difference lies in the fact that the interfacial energy transfer mainly occurs at a few atomic layers at the interface and thus leads to less competition with Mn^2+^ in trapping the excitation energy from excited Gd^3+^ ions^48^.

To enrich the color diversity of the short-lived upconversion emission, we further doped Er^3+^ ions into the nanoparticles (Fig. 3c). An additional layer of NaYF_4_ was used to separate the core and the shell comprising the Er^3+^ ions. The emission dependence of Er^3+^ on the Yb^3+^ content provides a precise control over the emission profile of the resultant nanoparticles. For example, intense white, green, or red color can be readily generated by varying the ratio of Yb^3+^ to Er^3+^ ions (5/0.05, 20/2 or 50/0.05 mol%) that are encapsulated in the outmost shell of the nanoparticles (Fig. 3d and Supplementary Fig. 15)^49^. In these cases, the emission profiles of the long-lived Mn^2+^ luminescence become indistinguishable due to the spectral overlap with the emission bands of Er^3+^ at 520 and 540 nm.

Remarkably, the distinct upconversion emission of Mn^2+^ in green could be clearly visualized after a short time interval when all lanthanide emissions ceased. This supports the lifetime measurements that the decay kinetics of Mn^2+^ emission is almost irrespective to the epitaxially grown shell of NaYF_4_:Yb/Er in presence of an inert shell layer of NaYF_4_ (Supplementary Fig. 16). Control experiments showed that multilayer nanoparticles without the NaYF_4_ layer generate merely a yellow emission from the lanthanides, but not the long-lasting luminescence from Mn^2+^ (Supplementary Fig. 17). This observation is likely due to the preferential energy transfer from Yb^3+^ → Er^3+^ or caused by Yb^3+^–Yb^3+^ energy migration from the inner layer of NaGdF_4_:Yb/Tm(49/1 mol%) to the outer layer of NaYF_4_:Yb/Er.

### Generation of binary temporal codes under 808 nm excitation

An efficient energy transfer from Nd^3+^ to Yb^3+^ ions allows the extension of the excitation wavelength of Mn^2+^-doped nanoparticles from 980 to 808 nm^50–52^. This was validated through an epitaxial growth of NaYF_4_:Nd(20 mol%) on NaGdF_4_:Mn(30 mol%)@NaGdF_4_:Yb/Tm(49/1 mol%) core-shell nanoparticles (Fig. 3e and Supplementary 18a). The excitation energy is first harvested by Nd^3+^ ions followed by energy transfer to Yb^3+^ and then energy relay through Yb^3+^ → Tm^3+^ → Gd^3+^ → Mn^2+^. The NaYF_4_:Nd(20 mol%) shell layer also supports Mn^2+^ upconversion emission under excitation at 980 nm (Supplementary Fig. 18b). Notably, the core-shell-shell nanoparticles of its cubic equivalent could not generate Mn^2+^ upconversion emission under laser irradiation at 808 nm (Supplementary Fig. 19).

To shed more light on creating short-lived color codes for Mn^2+^-doped nanoparticles under both 808 and 980 nm excitations, we investigated NaGdF_4_:Mn(30 mol%)@NaGdF_4_:Yb/Tm(49/1 mol%)@NaYF_4_:Nd(20 mol%) nanoparticles involving energy relay through Nd^3+^ → Yb^3+^ → Er^3+^ (Fig. 3e). We found that the epitaxial growth of NaYF_4_:Nd/Yb/Er with different dopant ratios onto NaGdF_4_:Mn@NaGdF_4_:Yb/Tm@NaYF_4_:Nd nanoparticles can lead to a marked change in color output, while retaining long-lived emission nature of Mn^2+^ (Fig. 3f and Supplementary Fig. 20). Note that the excitation of both Tm^3+^ and Mn^2+^ ions at 808 nm can be largely suppressed by introducing an inert layer of NaYF_4_ between NaGdF_4_:Yb/Tm(49/1 mol%) and NaYF_4_:Nd(20 mol%). This rendered the as-prepared multilayer nanoparticles with a white color output on 980 nm excitation, but a dominant yellow emission on 808 nm excitation (Supplementary Fig. 21).

### Multilevel anti-counterfeiting application

**Fig. 4:**
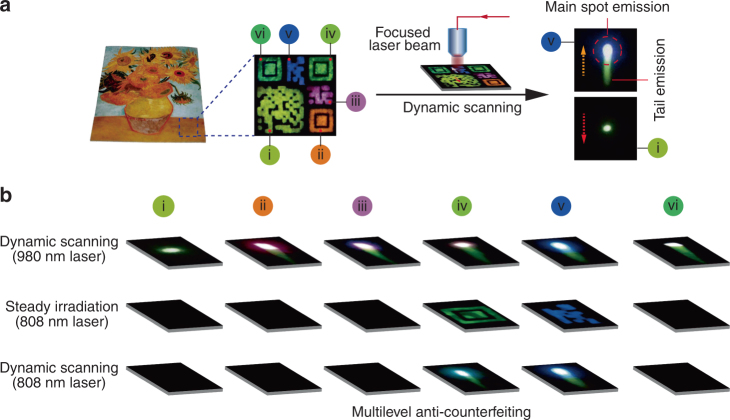
Multilevel anti-counterfeiting application with Mn^2+^-activated core-shell nanoparticles. **a** General design of the 2D patterns (i to vi) made with nanoparticles of different composition. Steady irradiation with a 980 nm laser (6 W cm^−2^) leads to multicolor features of the patterns, while dynamic scanning of the patterns with a focused laser beam (64 W cm^−2^) gives rise to a different scenario. Unlike purely lanthanide-doped nanoparticles with only a bright spot of emission emerging from pattern i under the dynamic scanning, the as-prepared Mn^2+^-doped nanoparticles show a bright spot of emission with a *green-colored tail* from pattern v. **b** Emission profiles of the patterns recorded under different irradiation conditions. Each pattern varies significantly in emission color under dynamic scanning at 980 nm. Steady irradiation or dynamic scanning at 808 nm of the patterns iv and v, made with Nd^3+^-sensitized nanoparticles, can provide a similar level of readout to that on 980 excitation. Nanoparticles used for generating the patterns: i NaYF_4_:Yb/Er (20/2 mol%); ii NaGdF_4_:Mn (30 mol%)@NaGdF_4_:Yb/Tm (49/1 mol%)@NaYF_4_@NaYF_4_:Yb/Er (50/0.05 mol%); iii NaGdF_4_:Mn (30 mol%)@NaGdF_4_:Yb/Tm(49/1 mol%)@NaYF_4_:Eu(20 mol%); iv NaGdF_4_:Mn(30 mol%)@NaGdF_4_:Yb/Tm(49/1 mol%)@NaYF_4_:Nd(20 mol%)@NaYF_4_:Nd/Yb/Er(2/10/1 mol%); v NaGdF_4_:Mn(30 mol%)@NaGdF_4_:Yb/Tm(49/1 mol%)@NaYF_4_:Nd(20 mol%); and vi NaGdF_4_:Mn(30 mol%)@NaGdF_4_:Yb/Tm(49/1 mol%)@NaYF_4_@NaYF_4_:Yb/Er(5/0.05 mol%)

The ability of our multilayer nanoparticles to simultaneously exhibit short- and long-lived emissions under excitation at either 980 or 808 nm offers a new class of optical materials ideal for multilevel authentication against product counterfeiting. As a proof-of-concept experiment, we made two-dimensional (2D) covert patterns on a reproduced artwork by stamping Mn^2+^-doped core-shell nanoparticles of different composition (Fig. 4a). As a control, colloidal NaYF_4_:Yb/Er(20/2 mol%) nanoparticles were also synthesized for the preparation of conventional upconversion anti-counterfeiting ink (Supplementary Fig. 22).

We found that a steady irradiation with a 980 nm laser (6 W cm^−2^) leads to multicolor patterns displaying characteristic emissions of lanthanide and Mn^2+^ emitting ions (Fig. 4a). Interestingly, dynamic scanning of the same patterns with a focused laser beam (at 64 W cm^−2^) yielded different emission patterns (Fig. 4b). For instance, only a main green spot of emission emerged from pattern i made with NaYF_4_:Yb/Er(20/2 mol%) nanoparticles, while an additional tailed emission in green was observed from other patterns (ii to vi, Supplementary Movie 1). More importantly, the color output of the main spot emission can be varied from red (pattern ii) to white (pattern vi) by controlling the doping composition of the lanthanides. As an added benefit, steady irradiation or dynamic scanning at 808 nm of the patterns iv and v made with Nd^3+^-sensitized nanoparticles resulted in emission features that are comparable to those obtained on 980 nm excitation.

It is important to note that the binary color codes with a tailed emission are difficult to obtain by excitation of a pattern comprising a simple mixture of NaYF_4_:Yb/Tm(20/0.2 mol%) nanoparticles and SrAl_2_O_4_:Eu^2+^/Dy^3+^ afterglow material. Although a blue upconversion emission can be observed from the pattern upon 980 nm excitation, dynamic laser scanning of the same area only offered a main spot of Tm^3+^ emission (Supplementary Fig. 23a, b). This observation suggests a weak energy transfer from the upconversion nanoparticles to the afterglow materials under investigation. This hypothesis is confirmed by the result that ultraviolet irradiation is more effective in generating a yellow-green afterglow signal relative to indirect excitation with a 980 nm laser (Supplementary Fig. 23c, d). Taken together, these results confirm that as-prepared Mn^2+^-doped upconversion nanoparticles are particularly promising for multilevel anti-counterfeiting applications without the need for time-gated set-up to separate and decode security data.

## Discussion

Our findings provide a new design for the creation of binary upconversion colors with two distinct timescales, which can be harnessed for data storage and security applications. This capability is enabled by combining a long-lived Mn^2+^ luminescence and short-lived lanthanide emission at the single particle level through core-shell engineering. Considering the advantages associated with 808 nm excitation for deep tissue penetration and with a concomitant long-lived luminescence, the advent of these Mn^2+^-doped nanoparticles may also have important implications for better in vivo cell tracking. Lastly, our synthetic strategy may offer a new, general route to prepare upconversion nanocrystals containing transition metal ions.

## Methods

### Preparation of Mn^2+^-doped multilayer nanocrystals

The Mn^2+^-doped hexagonal-phased NaGdF_4_ core nanoparticles were prepared through a hydrothermal method. Epitaxial growth of NaGdF_4_:Yb/Tm(49/1 mol%) and NaYF_4_ or NaYF_4_: Nd (Eu, Eu/Tb or Tb) onto the as-prepared core nanoparticles was enabled by a combination of the coprecipitation and thermal decomposition methods. Detailed experimental procedure for the preparation of different types of Mn^2+^-doped nanoparticles is provided in the Supplementary Methods.

### Preparation of ink solutions

The as-prepared nanoparticles were treated with in a mixed solution of ethanol (0.5 mL) and HCl (0.5 mL, 2 M) to remove surface ligands^53^. The ligand-free nanoparticles were collected by centrifugation at 16,500 rpm for 20 min, and were dispersed into a mixed solution of water (0.1 mL) and dimethyl sulfoxide (0.1 mL). For preparation of security ink solution containing both lanthanide-doped upconversion nanoparticles and afterglow luminescent materials, the as-prepared dispersion of NaYF_4_:Yb/Tm nanoparticles was added into a plastic tube (2.0 mL) charged with SrAl_2_O_4_:Eu^2+^/Dy^3+^ (10 mg). The mixture was sonicated for 15 min prior to use.

### Instrumentation

TEM measurement was carried out on a field emission transmission electron microscope (JEOL-JEM 2010F) operated at an acceleration voltage of 200 kV. Powder X-ray diffraction patterns were recorded on a Bruker D8 Advance diffractometer using graphite-monochromatized CuKα radiation (*λ* = 1.5406 Å). Luminescence spectra were measured with a DM150i monochromator equipped with a R928 photon-counting photomultiplier tube, in conjunction with a 980-nm diode laser. The decay curves were measured with a customized phosphorescence lifetime spectrometer (FSP920-C, Edinburgh), equipped with a digital oscilloscope (TDS3052B, Tektronix) and a tunable mid-band OPO laser as an excitation source (410–2400 nm, Vibrant 355II, OPOTEK).

### Data availability

The authors declare that the data that support the findings of this study are available within the article and its Supplementary Information files. All other relevant data are available from the corresponding author upon request.

## Electronic supplementary material

Supplementary Information

Description of Additional Supplementary Files

Supplementary Movie 1
