# Supplementary material for: Binary temporal upconversion codes of Mn2+-activated nanoparticles for multilevel anti-counterfeiting
Source: Nat Commun. 2017 Oct 12;8:899. doi: 10.1038/s41467-017-00916-7 (PMC5638907; doi:10.1038/s41467-017-00916-7)
Supplement: Supplementary file 1 — Supplementary Information [file 41467_2017_916_MOESM1_ESM.pdf]

Supplementary Figures

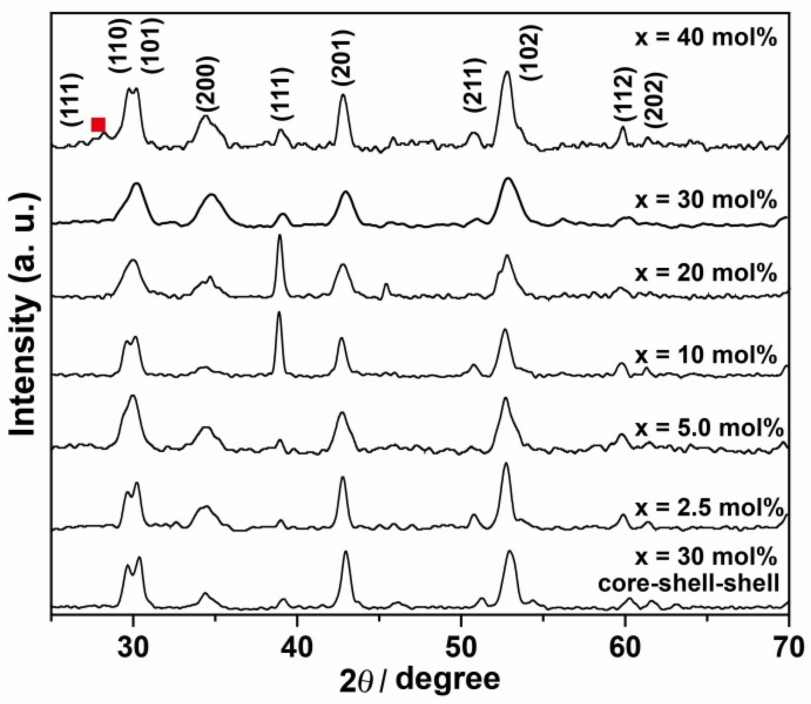

Supplementary Figure 1. XRD patterns of the as-prepared $NaGdF_4$:Mn (x mol%) (x: 2.5-40 mol%) core nanoparticles and $NaGdF_4$:Mn(30 mol%)@$NaGdF_4$:Yb/Tm(49/1 mol%)@$NaYF_4$ core-shell-shell nanoparticles. Diffraction peak corresponding to cubic phase is marked with a square box in red, indicating the occurrence of phase separation at a high Mn^{2+} doping content (40 mol%).

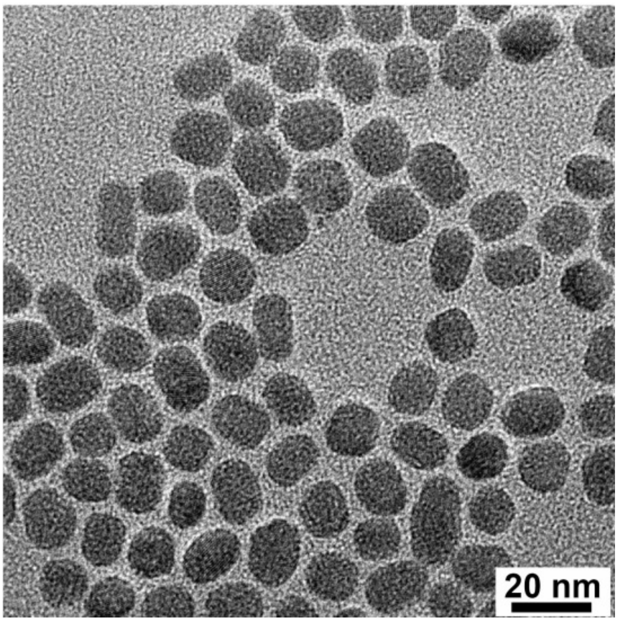

Supplementary Figure 2. TEM image of the as-prepared $NaGdF_4$:Mn (30 mol%) nanoparticles prepared by the hydrothermal method.

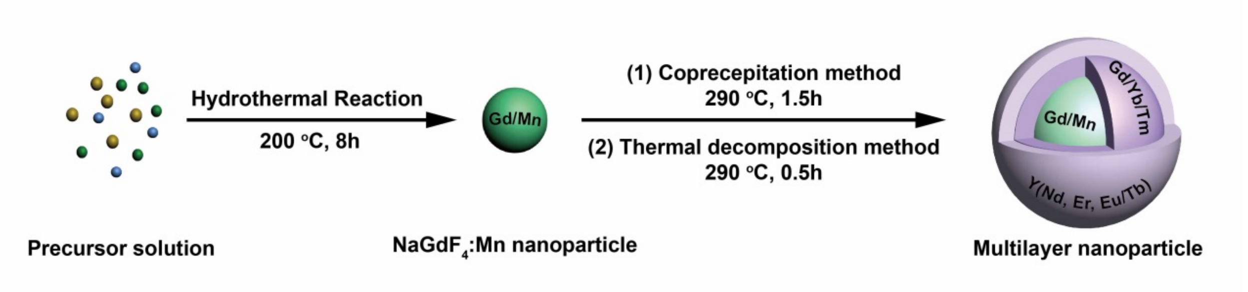

Supplementary Figure 3. The procedures used for the preparation of hexagonal-phased $NaGdF_4$:Mn@$NaGdF_4$:Yb/Tm@$NaYF_4$ or $NaGdF_4$:Mn@$NaGdF_4$:Yb/Tm@$NaYF_4$:A (A： Nd, Eu, Eu/Tb or Tb) multilayer nanoparticles.

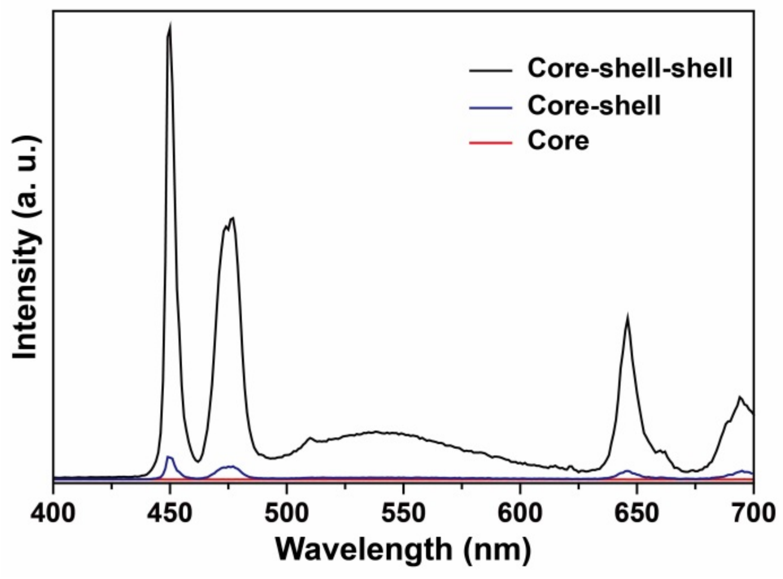

Supplementary Figure 4. Emission profiles of hexagonal-phased $NaGdF_4$:Mn (30 mol%) core nanoparticles, $NaYF_4$:Mn(30 mol%)@$NaGdF_4$:Yb/Tm (49/1 mol%) core-shell nanoparticles and $NaYF_4$:Mn(30 mol%)@$NaGdF_4$:Yb/Tm(49/1 mol%)@$NaYF_4$ core-shell-shell nanoparticles on excitation at 980 nm.

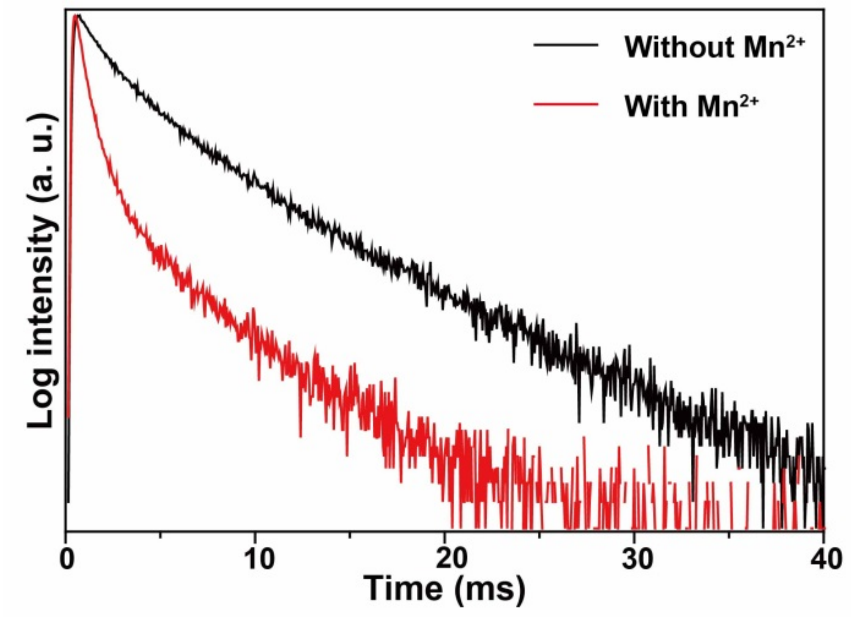

Supplementary Figure 5. A comparison of upconversion lifetimes of Gd^{3+} at 311 nm in multilayer nanoparticles of $NaGdF_4$@$NaGdF_4$:Yb/Tm(49/1 mol%)@$NaYF_4$ and $NaGdF_4$:Mn(30 mol%)@$NaGdF_4$:Yb/Tm(49/1 mol%)@$NaYF_4$.

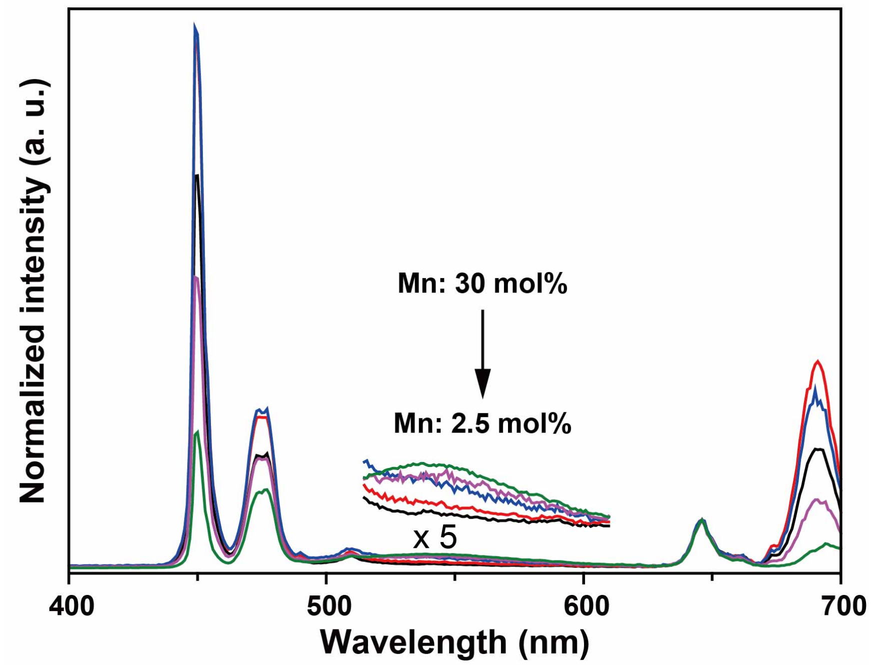

Supplementary Figure 6. Emission profiles of $NaGdF_4$:Mn (x mol%) (x: 2.5, 5, 10, 20 and 30 mol%)@$NaGdF_4$:Yb/Tm(49/1 mol%)@$NaYF_4$ core-shell-shell nanoparticles under excitation at 980 nm. For emission intensity comparison, the emission profiles were normalized at 646 nm.

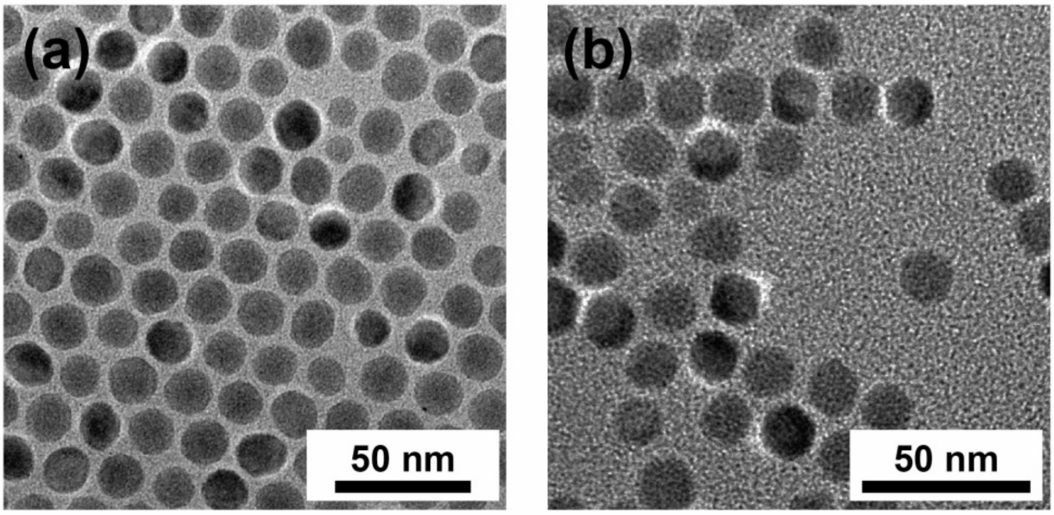

Supplementary Figure 7. Representative TEM images of (a) cubic-phased $NaGdF_4$:Yb/Tm(49/1 mol%)@$NaGdF_4$:Mn(64 mol%) core-shell nanoparticles and (b) cubic-phased $NaGdF_4$:Mn(30 mol%)@$NaGdF_4$:Yb/Tm (49/1 mol%)@$NaYF_4$ core-shell-shell nanoparticles.

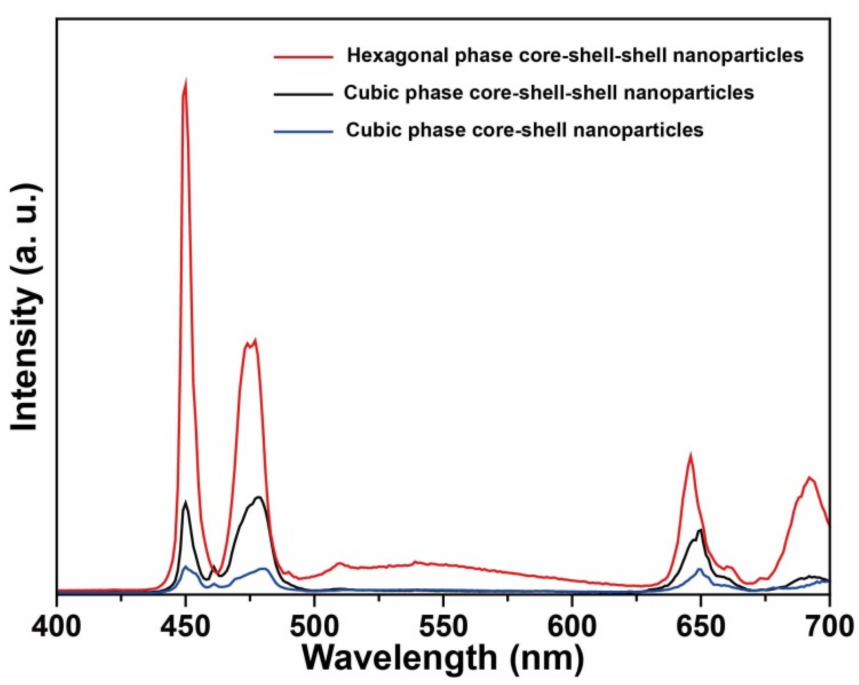

Supplementary Figure 8. A comparison of emission profiles of hexagonal-phased $NaGdF_4$:Mn(30 mol%)@$NaGdF_4$:Yb/Tm(49/1 mol%)@$NaYF_4$ core-shell-shell nanoparticles, cubic-phased $NaGdF_4$:Yb/Tm(49/1 mol%)@$NaGdF_4$:Mn(64 mol%) and cubic-phased $NaGdF_4$:Mn(30 mol%)@$NaGdF_4$:Yb/Tm(49/1 mol%)@$NaYF_4$ core-shell-shell nanoparticles under excitation at the same conditions (980 nm).

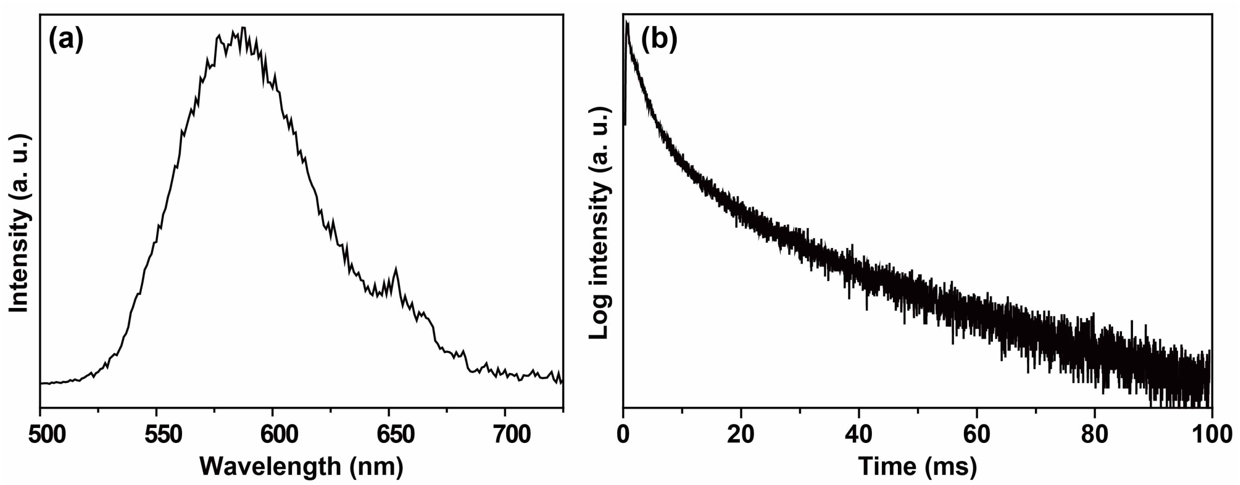

Supplementary Figure 9. (a) Emission profile $NaYF_4$:Yb/Mn (5/30 mol%) nanoparticles upon excitation at 980 nm. (b) Corresponding decay curve of upconversion emission of Mn^{2+} (580 nm, $^4T_1 \rightarrow {}^6A_1$) in as-prepared $NaYF_4$:Yb/Mn (5/30 mol%) nanoparticles.

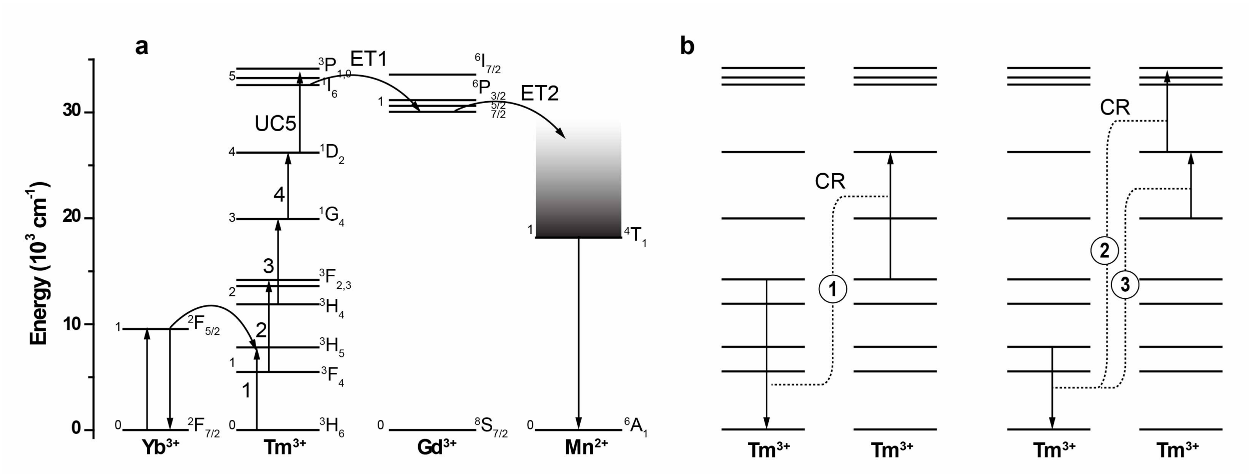

Supplementary Figure 10. (a) Proposed simplified mechanism demonstrating photon upconversion of Mn^{2+} ions in a nanoparticle platform. It can be divided into two three steps: i) photon upconversion of Tm^{3+} through a multi-photon upconversion process upon excitation at 980 nm, ii) Energy transfer from Tm^{3+} to Gd^{3+} and energy migration through Gd^{3+} ion arrays and iii) energy trapping by Mn^{2+} ions from the excited Gd^{3+} ions. (b). Proposed Tm^{3+}-Tm^{3+} cross-relaxation (CR) interactions occurred in the step of photon upconversion of Tm^{3+} ions.

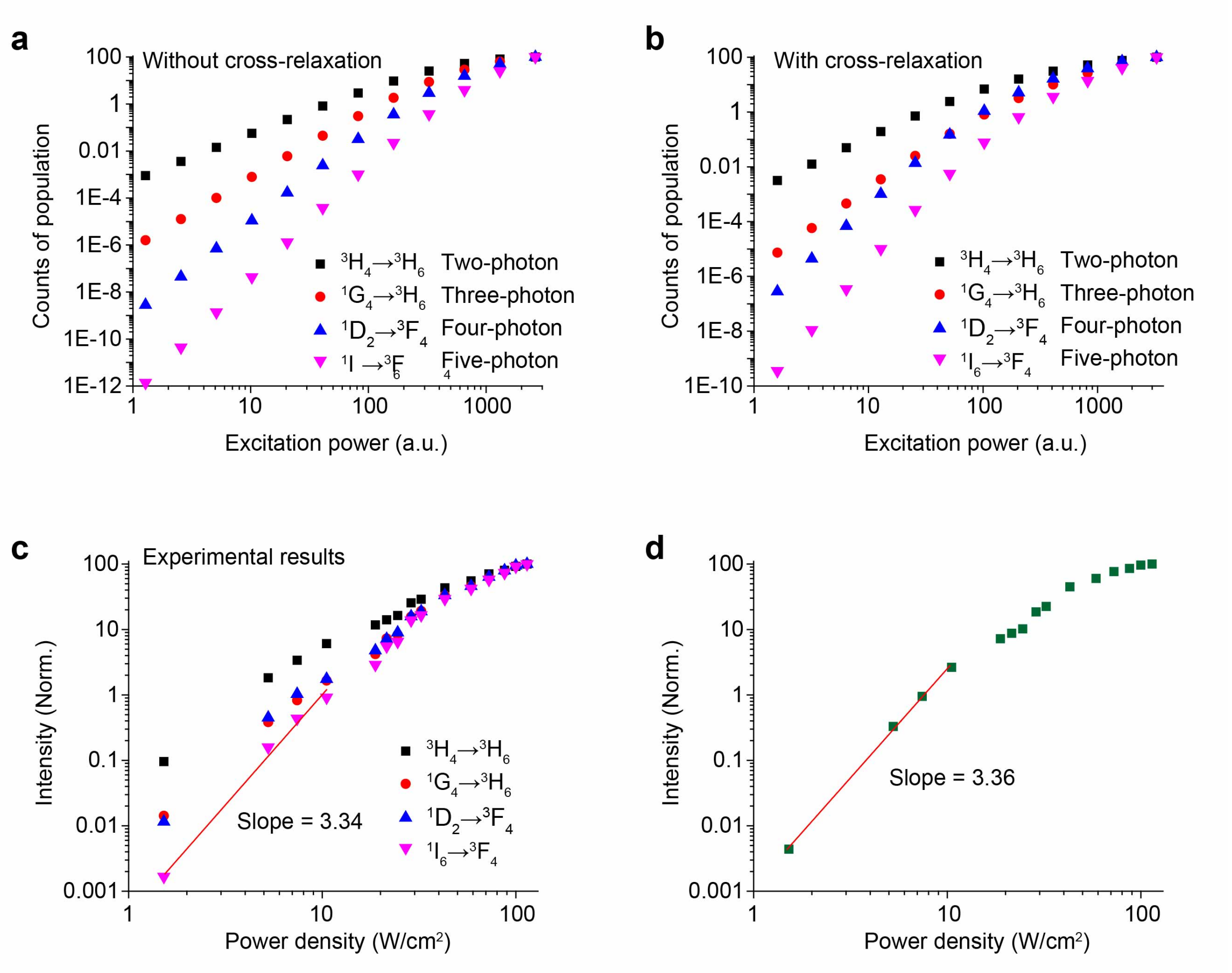

Supplementary Figure 11. (a, b) Simulated power dependence of Tm^{3+} emissions without and with consideration of Tm^{3+}-Tm^{3+} cross-relaxation interaction. (c, d), Experimental powder dependence of Tm^{3+} and Mn^{2+} emissions.

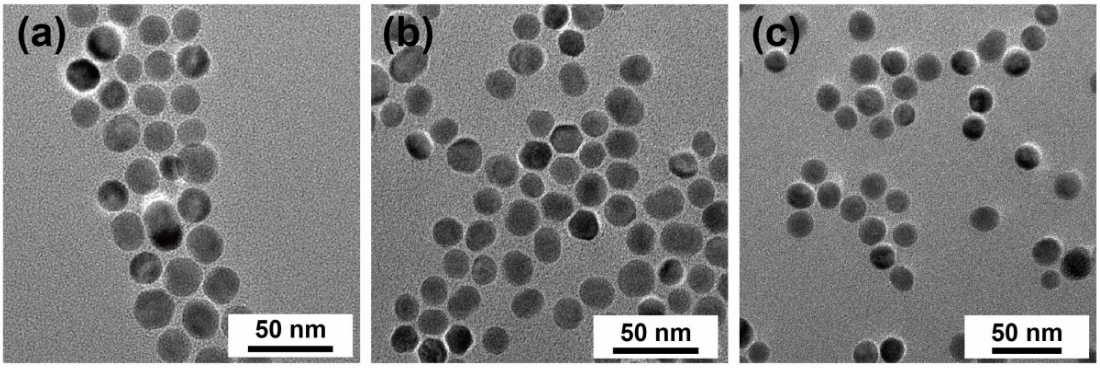

Supplementary Figure 12. Representative TEM images of hexagonal-phased $NaGdF_4$:Mn(30 mol%)@$NaGdF_4$:Yb/Tm (49/1 mol%)@$NaYF_4$:A (x mol%) core-shell-shell nanoparticles: (a) A = Eu^{3+}, x = 20; (b) A = Eu^{3+}/Tb^{3+}, x = 5/15; and (c) A = Tb^{3+}, x = 20.

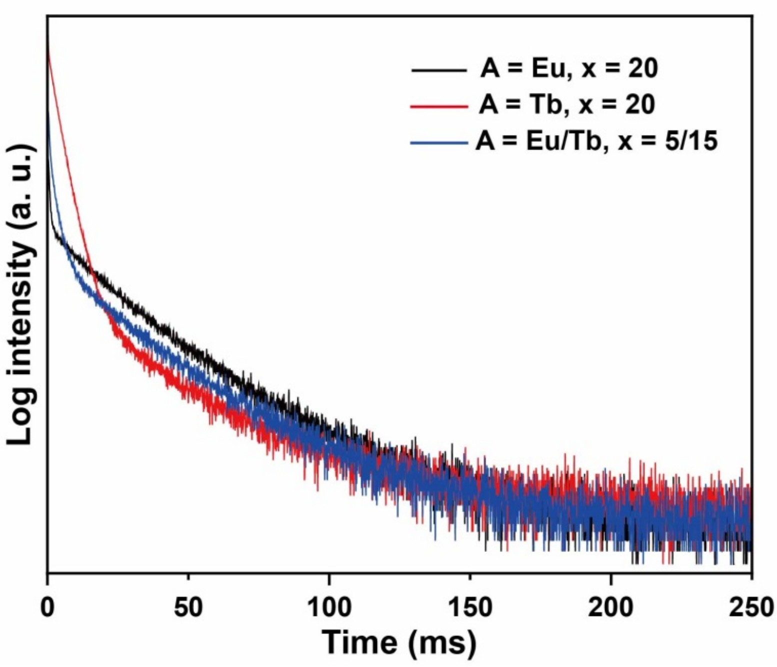

Supplementary Figure 13. Lifetimes of Mn^{2+} (550 nm, $^4T_1 \rightarrow ^6A_1$) measured in multilayer nanoparticles of $NaGdF_4$:Mn (30 mol%)@$NaGdF_4$:Yb/Tm(49/1 mol%)@$NaYF_4$:A (x mol%), A = Eu^{3+}, x = 20 (black curve); A = Tb^{3+}, x = 20 (red curve); and A = Eu^{3+}/Tb^{3+}, x = 5/15 (blue curve).

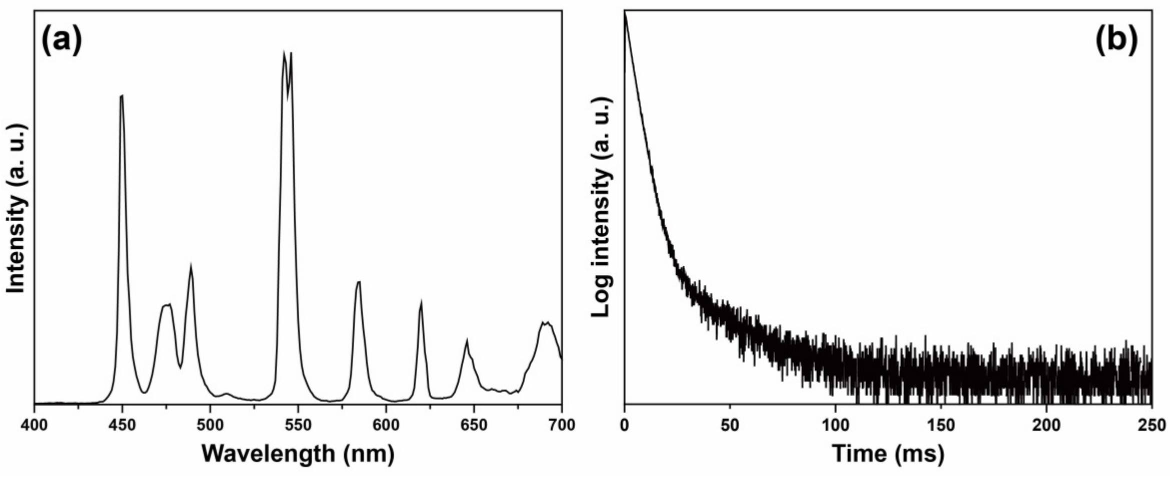

Supplementary Figure 14. (a) Emission profile of $NaGdF_4$:Mn(30 mol%)@$NaGdF_4$:Yb/Tm(49/1 mol%)@$NaGdF_4$:Tb (20 mol%) core-shell-shell nanoparticles. (b) Corresponding upconversion emission lifetime of Mn^{2+} (550 nm, $^4T_1 \rightarrow {}^6A_1$) in the as-prepared core-shell-shell nanoparticles.

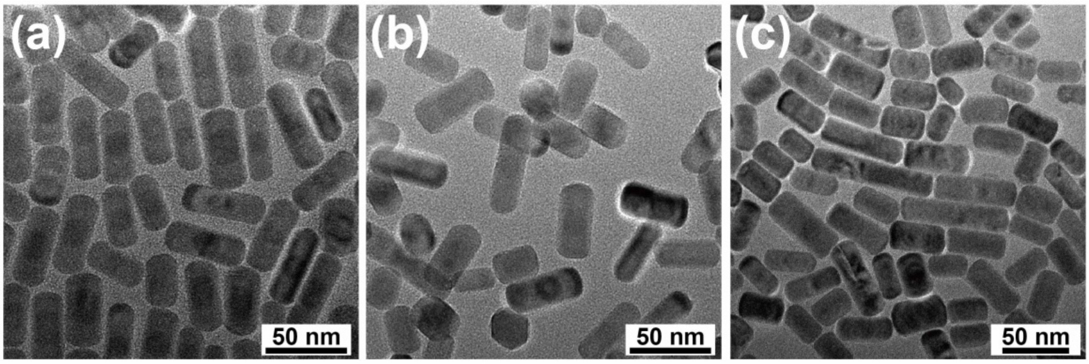

Supplementary Figure 15. Representative TEM images of multilayer nanoparticles of $NaGdF_4$:Mn(30 mol%)@$NaGdF_4$:Yb/Tm(49/1 mol%)@$NaYF_4$@$NaYF_4$:Yb/Er (x mol%): (a) x = 5/0.05; (b) x = 20/2; and (c) x = 50/0.05.

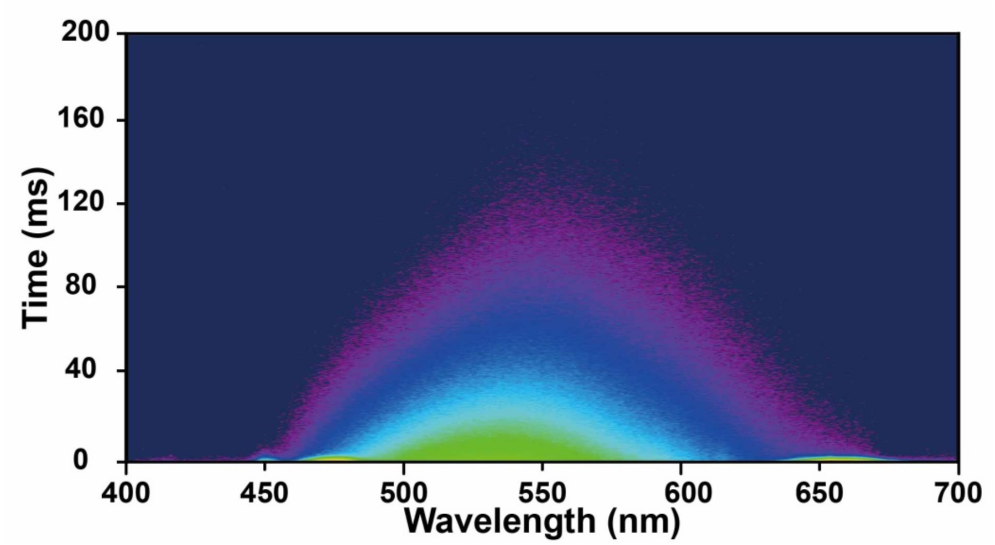

Supplementary Figure 16. Time-resolved emission spectra of multilayer nanoparticles of $NaGdF_4$:Mn(30 mol%)@$NaGdF_4$:Yb/Tm(49/1 mol%)@$NaYF_4$@$NaYF_4$:Yb/Er(50/0.05 mol%) measured at room temperature.

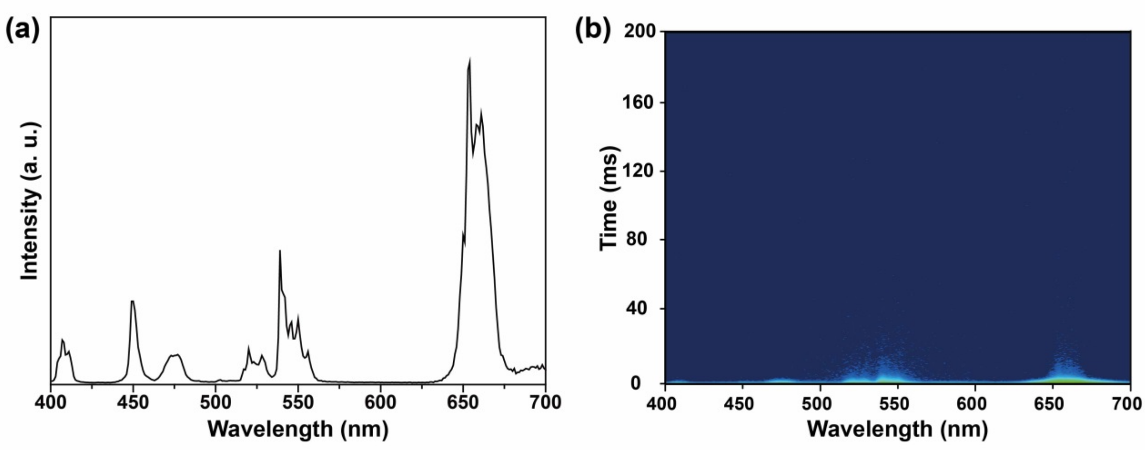

Supplementary Figure 17. (a) Emission profile and (b) Time-resolved emission spectra of the as-prepared multilayer nanoparticles of $NaGdF_4$:Mn(30 mol%)@$NaGdF_4$:Yb/Tm(49/1 mol%)@$NaYF_4$:Yb/Er(50/0.05 mol%) measured at room temperature.

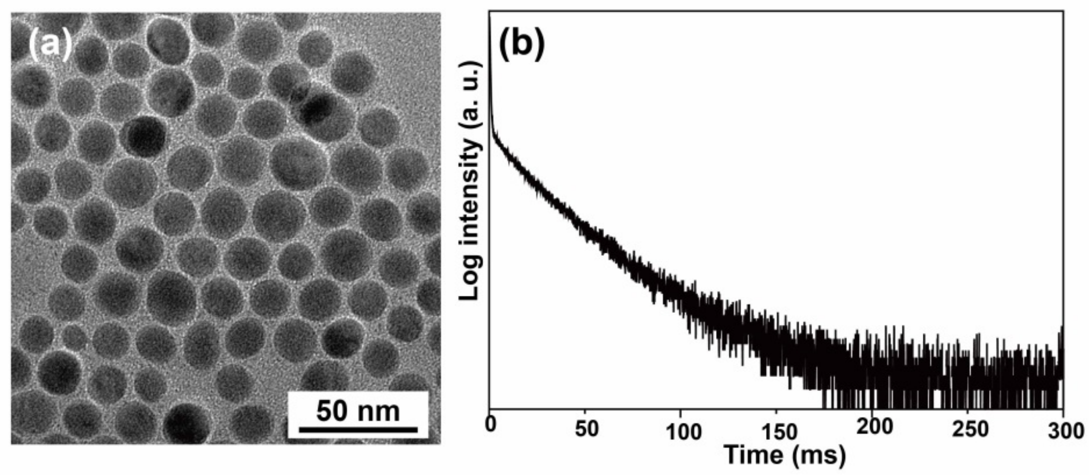

Supplementary Figure 18. (a) Representative TEM image of hexagonal-phased $NaGdF_4$:Mn(30 mol%)@$NaGdF_4$:Yb/Tm(49/1 mol%)@$NaYF_4$:Nd(20 mol%) core-shell-shell nanoparticles. (b) Corresponding lifetime of upconversion emission of Mn^{2+} measured at 550 nm ($^4T_1 \rightarrow {}^6A_1$).

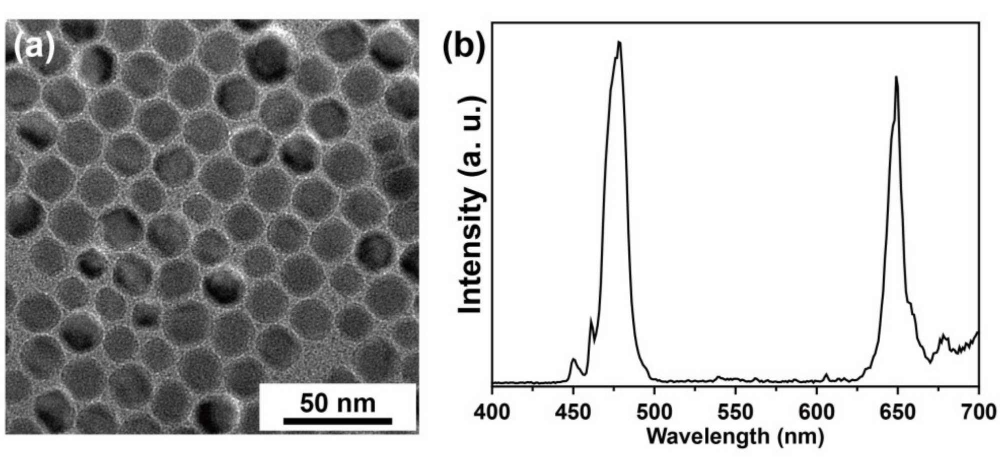

Supplementary Figure 19. (a) Representative TEM image and (b) the corresponding emission profile of cubic-phased $NaGdF_4$:Mn(30 mol%)@$NaGdF_4$:Yb/Tm(49/1 mol%)@$NaYF_4$:Nd(20 mol%) core-shell-shell nanoparticles.

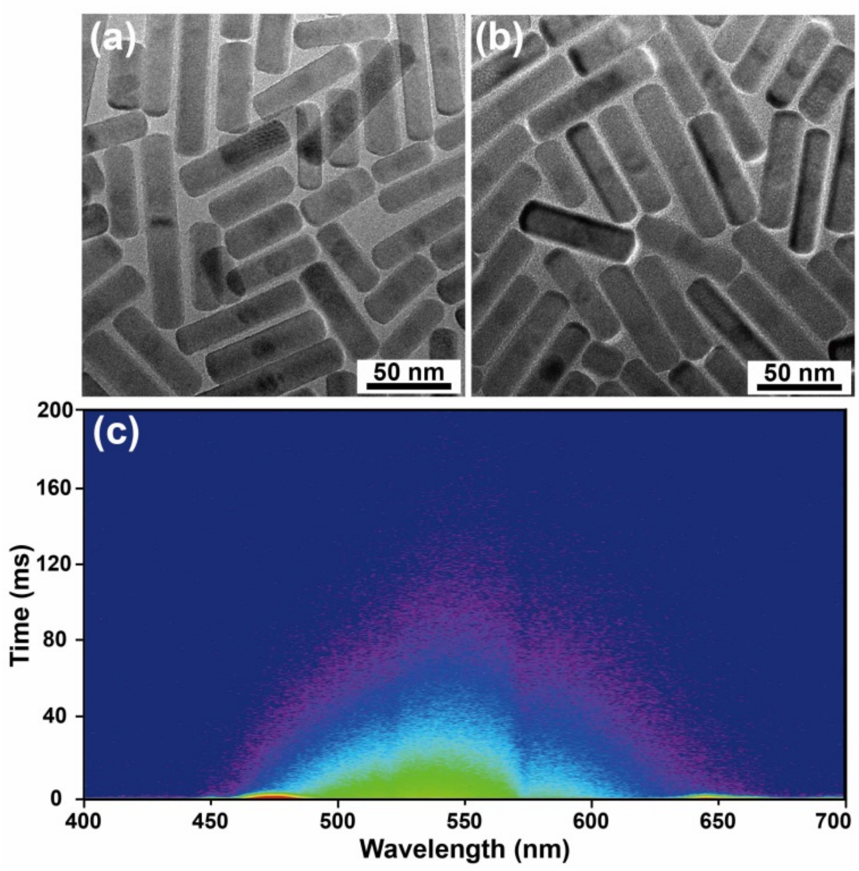

Supplementary Figure 20. (a, b) Representative TEM images of hexagonal-phased multilayer nanoparticles of $NaGdF_4$:Mn(30 mol%)@$NaGdF_4$:Yb/Tm(49/1 mol%)@$NaYF_4$:Nd(20 mol%)@$NaYF_4$:Nd/Yb/Er (x mol%): (a) x = 1/30/0.5 and (b) x = 2/10/1. (c) Time-resolved emission spectra of $NaGdF_4$:Mn(30 mol%)@$NaGdF_4$:Yb/Tm(49/1 mol%)@$NaYF_4$:Nd(20 mol%)@$NaYF_4$:Nd/Yb/Er(2/10/1 mol%) multilayer nanoparticles.

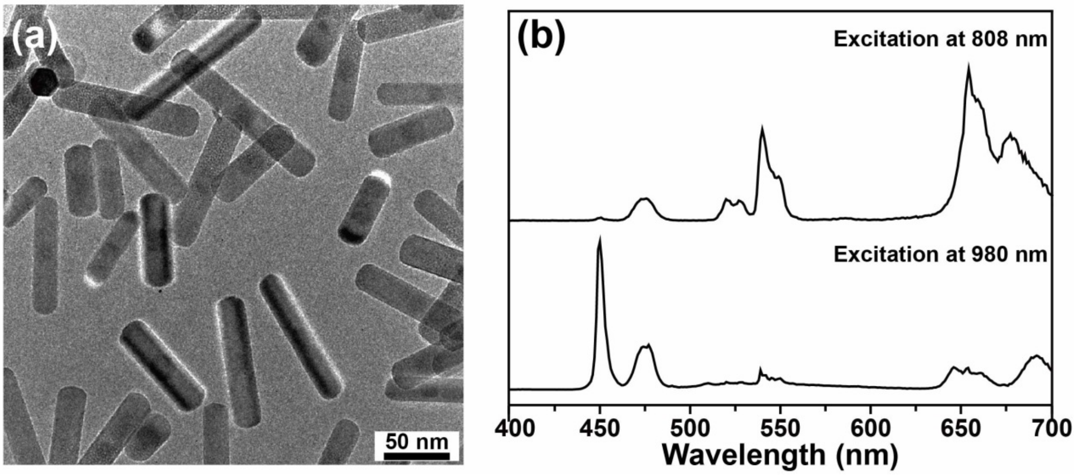

Supplementary Figure 21. (a) Representative TEM image of hexagonal-phased multilayer nanoparticles of $NaGdF_4$:Mn(30 mol%)@$NaGdF_4$:Yb/Tm(49/1 mol%)@$NaYF_4$@$NaYF_4$:Nd(20 mol%)@$NaYF_4$:Nd/Yb/Er (2/10/1 mol%). (b) The corresponding emission profiles of the as-prepared multilayer nanoparticles under excitation at 808 (top curve) and 980 nm (down curve).

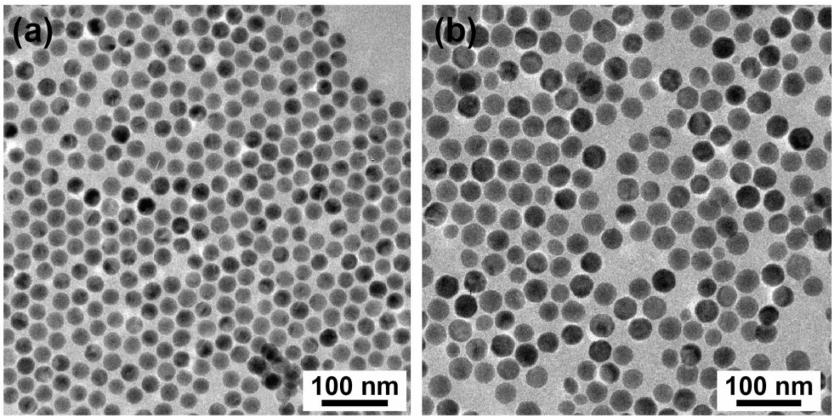

Supplementary Figure 22. Representative TEM images of hexagonal-phased $NaYF_4$:Yb/Er(20/2 mol%) and $NaYF_4$:Yb/Tm(20/0.2 mol%) nanoparticles prepared via the coprecipitation method.

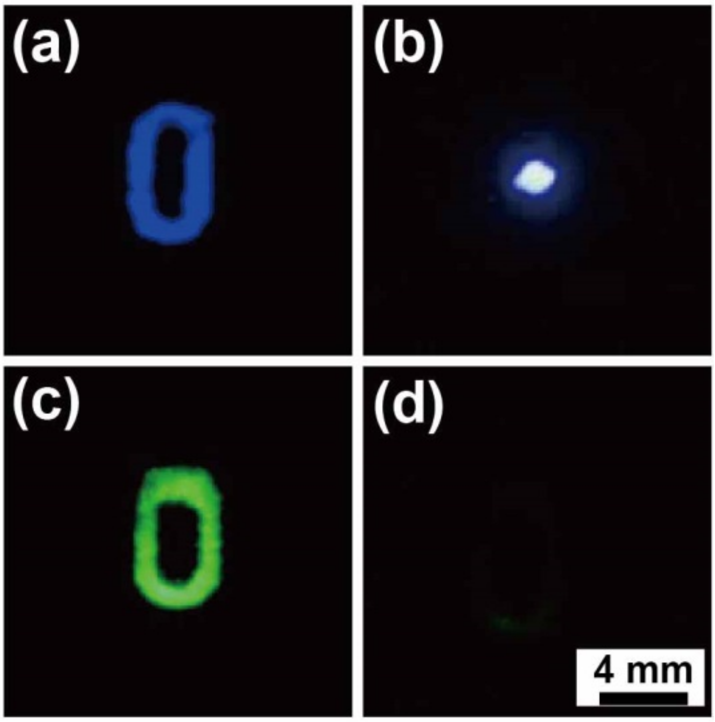

Supplementary Figure 23. (a, b) Upconversion Luminescence and dynamic scanning readout of pattern "O" made by using a mixture of $NaYF_4$:Yb/Tm(20/0.2 mol%) and afterglow luminescent material of $SrAl_2O_4$:Eu^{2+}/Dy^{3+} upon excitation at 980 nm. (c, d) Afterglow luminescent readout of the pattern after the removal of the excitation sources: (c) 365-nm UV lamp and (d) 980-nm diode laser.

Supplementary Table 1. A comparison of designed and measured concentrations of Mn^{2+} in the $NaGdF_4$:Mn core nanoparticles. Note that the doped concentration of Mn^{2+} was measured by ICP-AES after dissolving the nanoparticles in diluted HCl solution.

Designed molar ratio of Mn/Gd (%)	Measured molar ratio of Mn/Gd by ICP-AES (%)
2.5	0.043
5.0	0.168
10	0.213
15	0.276
30	0.278

Supplementary Methods

Materials. $GdCl_3$•xH_2O (99.9%), $MnCl_2$ (99.0%), $Gd(CH_3COO)_3$•xH_2O (99.9%), $Yb(CH_3COO)_3$•xH_2O (99.9%), $Tm(CH_3COO)_3$•xH_2O (99.9%), Ln_2O_3 (Ln = Y, Yb, Er, Tm, Eu and Tb, 99.9%), NaF (99.0%), NaOH (98+%), NH_4F (98+%), $NaCF_3COO$ (98%), 1-octadecene (ODE, 90%), oleic acid (OA, 90%) and oleylamine (OM, 75%) were purchased from Sigma-Aldrich and used as received without further treatment. Afterglow luminescent materials of $SrAl_2O_4$:Eu/Dy were obtained from Foshan Xiucai Chemicals Co., Ltd.

Synthesis of $Ln(CF_3COO)_3$ (Ln = Y, Yb, Tm, Eu and Tb) precursors. The lanthanide precursors were synthesized by using a literature procedure.[1] Typically, Ln_2O_3 (5 mmol) was first dissolved in an aqueous solution of trifluoroacetic acid (10 mL) at 80 °C, and the resulting transparent solution was then dried at 100 °C to afford the lanthanide trifluoroacetates in the form of powder. Thereafter, the as-prepared lanthanide trifluoroacetates were used to prepare corresponding lanthanide precursor solutions (0.2 M, 50 mL).

Synthesis of hexagonal-phased $NaGdF_4$: Mn (x mol%) (x: 2.5, 5, 10, 20, 30 and 40) nanoparticles: Hexagonal-phased Mn-doped $NaGdF_4$ nanoparticles were prepared by a well-established hydrothermal method.[2] Briefly, a mixture of OA (5 mL), ethanol (5 mL) and NaOH (0.2 g/mL, 1.2 mL) was first prepared at room temperature. An aqueous solution of $GdCl_3$ and $MnCl_2$ (2 mL, 0.4 mmol in total) was added into the mixture under vigorous stirring, and the resultant mixture was kept stirring for 15 min. Thereafter, another aqueous solution of NaF (2 M, 1.6 mL) was added into the mixture. After another 15 min, the mixture was transferred into a teflon-lined autoclave (16 mL) and subsequently heated at 200 °C for 8 h. The product was precipitated with ethanol, collected by centrifugation, washed with ethanol for several times and stored in cyclohexane (4 mL) for further use.

Synthesis of hexagonal-phased $NaGdF_4$: Mn (x mol%) (x: 2.5, 5, 10, 20, and 30)@$NaGdF_4$:Yb/Tm (49/1 mol%) core-shell nanoparticles: The core-shell nanoparticles were prepared by a seed-mediated procedure.[3] In a typical synthesis, shell precursor was first prepared by heating a mixture of $Ln(CH_3CO_2)_3$ (Ln = Gd, Yb and Tm, 0.2 mmol), OA (4.0 mL) and ODE (6.0 mL) at 150 °C for 1.5 h, and the as-prepared transparent colorless solution was cooled down to 80 °C. A cyclohexane dispersion of $NaGdF_4$: Mn (x mol%, x = 5, 10, 20, 30 and 40, 1.0 mL) was then added into the precursor solution, and the resultant mixture was kept at 80 °C for 30 min. When the reaction temperature was decreased to 50 °C, a mixed methanol solution (3 mL) of NaOH (0.5 mmol) and NH_4F (0.8 mmol) was added under

vigorous stirring. After 30 min, the mixture was heated at 100 ^{o}C for 30 min to remove volatile components in the mixture, and then was further heated at 290 ^{o}C for 1.5 h under argon protection. After cooling to room temperature, the product was collected by centrifugation after the addition of excessive ethanol into the reaction system. The precipitate was further washed with a mixture of ethanol and cyclohexane, and finally stored in cyclohexane (4 mL).

Synthesis of hexagonal-phased $NaGdF_4$:Mn (x mol%) (x: 2.5, 5, 10, 20, and 30 mol%)@$NaGdF_4$:Yb/Tm (49/1 mol%)@$NaYF_4$ multilayer nanoparticles: The multilayer nanoparticles were prepared on the basis of the procedure for making the above-mentioned core-shell nanoparticles. After the growth of the shell of $NaGdF_4$:Yb/Tm (49/1 mol%), a mixture of OA (1 mL), ODE (1 mL) containing $Na(CF_3COO)$ (0.2 mmol) and $Y(CF_3COO)_3$ (0.2 mmol) was injected at 290 ^{o}C, and the reaction system was maintained at this temperature for another 30 min. After cooling to room temperature, the product was precipitated by addition of ethanol, collected by centrifugation and washed with a mixture of ethanol and cyclohexane. The purified nanoparticles were stored in cyclohexane (4 mL).

Synthesis of hexagonal-phased $NaGdF_4$:Mn(30 mol%)@$NaGdF_4$:Yb/Tm(49/1 mol%)@$NaYF_4$: (Nd 20 mol%, Eu, 20 mol%, Eu/Tb, 15/5 mol% or Tb 20 mol%) multilayer nanoparticles: The synthetic procedure for the multilayer nanoparticles is essentially identical to that for $NaGdF_4$:Mn(30 mol%)@$NaGdF_4$:Yb/Tm(49/1 mol%)@$NaYF_4$ core-shell nanoparticles. First, precursor solutions for the outermost layer was prepared by heating a mixture of OA (1.0 mL), ODE (1.0 mL), $Na(CF_3COO)$ (0.2 mmol) and $Ln(CF_3COO)_3$ (0.2 mmol in total) at 100 ^{o}C for 30 min to remove volatile components. Upon completing the growth of $NaGdF_4$:Yb/Tm (49/1 mol%) on $NaGdF_4$: Mn (30 mol%) by the above-mentioned coprecipitation method, the as-prepared precursor solutions were directly injected at 290 ^{o}C. Note that there is no need for harvesting the core-shell nanoparticles of $NaGdF_4$: Mn(30 mol%)@$NaGdF_4$:Yb/Tm (49/1 mol%) before the growth of the third shell lattice. The as-prepared multilayer nanoparticles were also stored in cyclohexane (4 mL) after purification.

Synthesis of hexagonal-phased $NaGdF_4$:Mn(30 mol%)@$NaGdF_4$:Yb/Tm(49/1 mol%)@$NaYF_4$@: $NaYF_4$:Yb/Er(5/0.05, 20/2 or 50/0.05 mol%) multilayer nanoparticles: The multilayer nanoparticles were synthesized on the base of $NaGdF_4$:Mn(30 mol%)@$NaGdF_4$:Yb/Tm(49/1 mol%)@$NaYF_4$ core-shell-shell nanoparticles via a hydrothermal method. Typically, a cyclohexane dispersion (1 mL) of the core-shell-shell nanoparticles was added into a mixture of $LnCl_3$ (Ln = Y, Yb and Er, 0.2 M, 1 mL), OA (5 mL), ethanol (5 mL) and NaOH (0.2 g/mL, 1.5 mL) under stirring.

After 0.5h, an aqueous solution of NH_4F (2 M, 0.5 mL) was added into the mixture. After another 15 min, the resulting mixture was transferred into a teflon-lined autoclave (16 mL) and subsequently heated at 180 °C for 8 h.

Synthesis of hexagonal-phased $NaGdF_4$:Mn(30 mol%)@$NaGdF_4$:Yb/Tm(49/1 mol%)@$NaYF_4$:Nd(20 mol%)@$NaYF_4$:Nd/Yb/Er(1/30/0.5, 2/10/1 mol%) multilayer nanoparticles: The synthetic procedure for the multilayer nanoparticles is identical to that for $NaGdF_4$:Mn(30 mol%)@$NaGdF_4$:Yb/Tm(49/1 mol%)@$NaYF_4$@: $NaYF_4$:Yb/Er(5/0.05 mol%) multilayer nanoparticles except that $NaGdF_4$:Mn(30 mol%)@$NaGdF_4$:Yb/Tm(49/1 mol%)@$NaYF_4$:Nd(20 mol%) and $LnCl_3$ (Ln = Y, Nd, Yb and Er) were used as core and lanthanide precursor in the growth of the outmost shell.

Synthesis of hexagonal-phased $NaGdF_4$:Mn(30 mol%)@$NaGdF_4$:Yb/Tm(49/1 mol%)@$NaYF_4$ @$NaYF_4$:Nd(20 mol%)@$NaYF_4$:Nd/Yb/Er (2/10/1 mol%) multilayer nanoparticles: The synthesis of the multilayer nanoparticles was identical to that for $NaGdF_4$:Mn(30 mol%)@$NaGdF_4$:Yb/Tm(49/1 mol%) @$NaYF_4$@$NaYF_4$:Yb/Er(5/0.05, 20/2 or 50/0.05 mol%) core-shell-shell nanoparticles except that the epitaxial growth was repeated for two times to grow the two outmost layers. In the shell growth of $NaYF_4$:Nd(20 mol%) and $NaYF_4$:Nd/Yb/Er (2/10/1 mol%), $LnCl_3$ (Ln = Y and Nd) and $LnCl_3$ (Ln = Y, Nd, Yb and Er) were used as lanthanide precursors, respectively.

Preparation of hexagonal-phased $NaYF_4$:Yb/Tm(20/0.2 mol%) or $NaYF_4$:Yb/Er(18/2 mol%) nanoparticles. The upconversion nanoparticles were prepared by the coprecipitation method. Typically, a precursor solution was first prepared by heating a mixture of OA (1.0 mL), ODE (1.0 mL) and $Ln(CH_3CO_2)_3$ (Ln = Y, Yb and Er/Tm, 0.4 mmol in total) at 150 °C for 1.5 h. Next, a methanol solution (6 mL) of NaOH (1.0 mmol) and NH_4F (1.6 mmol) was added into the precursor solution, and the resulting mixture was kept stirring for 0.5 h. The mixture was further heated at 100 °C under vacuum for 0.5 h and finally heated at 290 °C for 1.5 h under the protection of argon. Upon cooling to room temperature, the product was collected by centrifugation at 6000 rpm for 10 min after adding an excessive amount of ethanol. After being washed with a mixture of ethanol and cyclohexane, the obtained nanoparticles were dispersed in cyclohexane (4 mL).

Preparation of cubic-phased core-shell nanoparticles of $NaGdF_4$:Yb/Tm@$NaGdF_4$:Mn. The cubic-phased core-shell were prepared according to our previously reported method.[4] First, core nanoparticles were prepared by thermal

treatment of $Ln(CF_3COO)_3$ (Ln = Gd, Yb, Tm, Gd/Yb/Tm = 50/49/1, 1.0 mmol in total) and $NaCF_3COO$ (1.0 mmol) at 310 °C for 0.5 h in a mixture of OA (3.2 mL), ODE (6.4 mL) and OM (3.2 mL) under protection of N_2. The core nanoparticles were obtained by centrifugation at 6000 rpm for 10 min after the addition of excessive amount of ethanol, washed with a mixture of cyclohexane and ethanol and stored in cyclohexane (5 mL). The as-prepared colloidal solution (1.0 mL) was added into a pre-prepared shell precursor solution of OA (5.0 mL), ODE (5.0 mL), $Gd(CF_3COO)_3$ (0.2 mmol), $NaCF_3COO$ (0.2 mmol) and $Mn(CF_3COO)_2$ (0.36 mmol). After removal of volatile components in the resulting mixture at 100 °C under vacuum, the reaction system was heated 310 °C for 0.5 h under a nitrogen atmosphere. The cores-shell nanoparticles were collected, washed and stored by the way used for the preparation of the core nanoparticles.

Synthesis of cubic-phases $NaGdF_4$:Mn@$NaGdF_4$:Yb/Tm@$NaYF_4$ (or $NaYF_4$:Nd) core-shell-shell nanoparticles. The core-shell-shell nanoparticles were also prepared by a thermal decomposition method. Core nanoparticles were synthesized by heating a mixture of OA (5.0 mL) and ODE (5.0 mL) containing $Gd(CF_3COO)_3$ (0.7 mmol), $Mn(CF_3COO)_2$ (0.3 mmol) and $NaCF_3COO$ (1.0 mmol) at 310 °C for 0.5 h. After cooling to room temperature, the core nanoparticles were then collected by centrifugation at 6000 rpm for 5 min after the addition of excessive amount of ethanol, washed with a mixture of cyclohexane and ethanol, and finally stored in 5.0 mL of cyclohexane. 1.0 mL of the as-prepared cyclohexane dispersion was introduced into a shell precursor solution of OA (5.0 mL) and ODE (5.0 mL), $Ln(CF_3COO)_3$ (Ln = Gd, Yb, Tm, Gd/Yb/Tm = 50/49/1, 0.4 mmol in total) and $NaCF_3COO$ (0.4 mmol). The resulting mixture was heated at 80 °C for 0.5 h to remove cyclohexane, and subsequently heated at 100 °C under vacuum for 0.5 h to remove volatile impurities in the mixture. The reaction system was further heated at 310 °C for 0.5 h to proceed the growth of the first shell. The core-shell nanoparticles were collected and purified by the same method used for treatment of the core nanoparticles. The purified core-shell nanoparticles were re-dispersed into 2.0 mL of cyclohexane. The procedure used for the growth of the second shell is the same as that for the first shell except that $Ln(CF_3COO)_3$ (Ln =Y or Y/Nd, Y/Nd = 80/20 mol%, 0.2 mmol in total) and $NaCF_3COO$ (0.2 mmol) are used as the shell precursor.

Preparation of ligand-free core-shell-shell nanoparticles. The as-prepared core-shell or core-shell-shell nanoparticles are required to remove surface ligands before the preparation of security ink solutions.[5] In a typical procedure, the nanoparticles were first precipitated by addition of ethanol (1.0 mL) to a cyclohexane colloidal solution of the

nanoparticles (0.5 mL) and then collected by centrifugation at 6000 rpm for 5 min. The obtained nanoparticles were re-dispersed in a mixed solution of ethanol (0.5 mL) and HCl (0.5 mL, 2 M) upon sonication for 5 min. The ligand-free nanoparticles were collected by centrifugation at 16500 rpm for 20 min and re-dispersed in deionized water (0.5 mL).

Computational details: The upconversion mechanism of the Mn^{2+} emission in $NaGdF_4$:Mn@$NaGdF_4$:Yb/Tm@$NaYF_4$ nanocrystals is proposed in Supplementary Figure 10. Upconversion rate equations are employed to describe the multi-step energy transfer processes. According to the proposed upconversion mechanism, the rate equations of each energy states can be derived as follows:

For Yb^{3+}

$$Yb^{3+}(^2F_{5/2}): \quad \frac{dn_{Yb1}}{dt} = \sigma_{Yb} I n_{Yb0} - w_{Yb1} n_{Yb1} - u_1 n_{Yb1} n_{Tm0} - u_2 n_{Yb1} n_{Tm1} - u_3 n_{Yb1} n_{Tm2} - u_4 n_{Yb1} n_{Tm3} - u_5 n_{Yb1} n_{Tm4} \qquad \text{Eqn. 1}$$

$$Yb^{3+}(^2F_{7/2}): \quad \frac{dn_{Yb0}}{dt} = -\frac{dn_{Yb0}}{dt} \qquad \text{Eqn. 2}$$

For Tm^{3+}

$$Tm^{3+}(^3H_6): \quad \frac{dn_{Tm0}}{dt} = w_{Tm1} n_{Tm1} + w_{Tm2} n_{Tm2} + w_{Tm3} n_{Tm3} + w_{Tm4} n_{Tm4} + w_{Tm5} n_{Tm5} + w_{ET1} n_{Tm5} n_{Gd0} + w_{CR1} n_{Tm2}^2 + w_{CR2} n_{Tm1} n_{Tm4} + w_{CR3} n_{Tm1} n_{Tm3} - u_1 n_{Yb1} n_{Tm0} \qquad \text{Eqn. 3}$$

$$Tm^{3+}(^3F_4,^3H_5): \quad \frac{dn_{Tm1}}{dt} = u_1 n_{Yb1} n_{Tm0} - w_{Tm1} n_{Tm1} - u_2 n_{Yb1} n_{Tm1} - w_{CR2} n_{Tm1} n_{Tm4} - w_{CR3} n_{Tm1} n_{Tm3} \qquad \text{Eqn. 4}$$

$$Tm^{3+}(^3H_4,^3F_{2,3}): \quad \frac{dn_{Tm2}}{dt} = u_2 n_{Yb1} n_{Tm1} - w_{Tm2} n_{Tm2} - u_3 n_{Yb1} n_{Tm2} - 2 w_{CR1} n_{Tm2}^2 \qquad \text{Eqn. 5}$$

$$Tm^{3+}(^1G_4): \quad \frac{dn_{Tm3}}{dt} = u_3 n_{Yb1} n_{Tm2} - w_{Tm3} n_{Tm3} - u_4 n_{Yb1} n_{Tm3} - w_{CR3} n_{Tm1} n_{Tm3} \qquad \text{Eqn. 6}$$

$Tm^{3+}(^1D_2)$: $$\frac{dn_{Tm4}}{dt} = u_4 n_{Yb1} n_{Tm3} + w_{CR1} n_{Tm2}^{\ 2} + w_{CR3} n_{Tm1} n_{Tm3} - w_{Tm4} n_{Tm4} - u_5 n_{Yb1} n_{Tm4} - w_{CR2} n_{Tm1} n_{Tm4}$$ Eqn. 7

$Tm^{3+}(^1I_6, ^3P_{1,0})$: $$\frac{dn_{Tm5}}{dt} = u_5 n_{Yb1} n_{Tm4} + w_{CR2} n_{Tm1} n_{Tm4} - w_{Tm5} n_{Tm5} - w_{ET1} n_{Tm5} n_{Gd0}$$ Eqn. 8

For Gd^{3+}

$Gd^{3+}(^6P_{3/2,5/2,7/2}, ^6I_{7/2})$: $$\frac{dn_{Gd1}}{dt} = w_{ET1} n_{Tm5} n_{Gd0} - w_{Gd1} n_{Gd1} - w_{ET2} n_{Gd1} n_{Mn0}$$ Eqn. 9

$Gd^{3+}(^8S_{7/2})$: $$\frac{dn_{Gd0}}{dt} = -\frac{dn_{Gd0}}{dt}$$ Eqn. 10

For Mn^{2+}

$Mn^{2+}(^4T_1)$: $$\frac{dn_{Mn1}}{dt} = w_{ET2} n_{Gd1} n_{Mn0} - w_{Mn1} n_{Mn1}$$ Eqn. 11

$Mn^{2+}(^6A_1)$: $$\frac{dn_{Mn0}}{dt} = -\frac{dn_{Mn1}}{dt}$$ Eqn. 12

Where n_{Ybi}, n_{Tmi}, n_{Gdi}, n_{Mni} and w_{Ybi}, w_{Tmi}, w_{Gdi}, w_{Mni} (i = 0, 1 for Yb, Gd, Mn; or 0 to 5 for Tm) represent the population densities and the intrinsic decay rates of the corresponding energy states of Yb^{3+}, Tm^{3+}, Gd^{3+} and Mn^{2+} ions, respectively. u_i (i = 1 to 5) is the upconversion energy transfer rate from the Yb^{3+} to Tm^{3+} during the one-, two-, three-, four-, five-photon processes, respectively. w_{ET1} and w_{ET2} represent the energy transfer from Tm^{3+} to Gd^{3+} and from Gd^{3+} to Mn^{2+}, respectively. w_{CR1}, w_{CR2} and w_{CR3} represent the cross-relaxation processes [$^3F_{2,3}$ (Tm^{3+}) + $^3F_{2,3}$ (Tm^{3+}) $\longrightarrow$ $^1D_2$ (Tm^{3+}) + $^3H_6$ (Tm^{3+})], [$^3H_5$ (Tm^{3+}) + $^1D_2$ (Tm^{3+}) $\longrightarrow$ $^1I_6$ (Tm^{3+}) + $^3H_6$ (Tm^{3+})] and [$^3H_5$ (Tm^{3+}) + $^1G_4$ (Tm^{3+}) $\longrightarrow$ $^1D_2$ (Tm^{3+}) + $^3H_6$ (Tm^{3+})], respectively. $\sigma_{Yb} I$ refers to the excitation rate of Yb^{3+}, and I refers to the excitation power density.

The Eqns. 1-S12 describe the changes in the population density of each energy state in $NaGdF_4$:Mn@$NaGdF_4$:Yb/Tm@$NaYF_4$ nanocrystals as a function of excitation time (t). Considering a steady-state

condition under a constant irradiance at which time the population and deactivation of the excited energy state in the nanocrystals exists equilibrium as defined by

$$\frac{dn_{Ybi}}{dt} = \frac{dn_{Tmi}}{dt} = \frac{dn_{Gdi}}{dt} = \frac{dn_{Mni}}{dt} = 0 \qquad \text{Eqn. 13}$$

When the nanocrystals are excited by a low photon fluence, the intrinsic decay ($w_{Tmi}n_{Tmi}$) at the intermediate states of Tm^{3+} should be much larger than the upconversion energy transfer ($u_i n_{Yb1} n_{Tmi}$) and the Tm-Tm cross-relaxation ($w_{CRi} n_{Tmj} n_{Tmk}$). Herein, the density of each energy state obeys the classical upconversion rule as

$$n_{Tm1} \approx \frac{u_1 n_{Tm0}}{w_{Tm1}} n_{Yb1} \in n_{Yb1} \in I \qquad \text{Eqn. 14}$$

$$n_{Tm2} \approx \frac{u_1 u_2 n_{Tm0}}{w_{Tm1} w_{Tm2}} n_{Yb1}{}^2 \in n_{Yb1}{}^2 \in I^2 \qquad \text{Eqn. 15}$$

$$n_{Tm3} \approx \frac{u_1 u_2 u_3 n_{Tm0}}{w_{Tm1} w_{Tm2} w_{Tm3}} n_{Yb1}{}^3 \in n_{Yb1}{}^3 \in I^3 \qquad \text{Eqn. 16}$$

$$n_{Tm4} \approx \frac{u_1 u_2 u_3 u_4 n_{Tm0}}{w_{Tm1} w_{Tm2} w_{Tm3} w_{Tm4}} n_{Yb1}{}^4 \in n_{Yb1}{}^4 \in I^4 \qquad \text{Eqn. 17}$$

$$n_{Tm5} \approx \frac{u_1 u_2 u_3 u_4 u_5 n_{Tm0}}{w_{Tm1} w_{Tm2} w_{Tm3} w_{Tm4} (w_{Tm5} + w_{ET1} n_{Gd0})} n_{Yb1}{}^5 \in n_{Yb1}{}^5 \in I^5 \qquad \text{Eqn. 18}$$

and

$$n_{Mn1} \in n_{Gd1} \in n_{Tm5} \in n_{Yb1}{}^5 \in I^5 \qquad \text{Eqn. 19}$$

However, in real situation at a higher photon fluence the contribution of upconversion and cross-relaxation would clearly lower the exponential dependence of the density of states. Given that the energy transfer rate w_i, upconversion rate u_i and the amount of lanthanide ions n are constant for a given nanocrystal, it is possible for us to calculate the population densities of each energy state as a function of excitation power (I) with a simulation method using Mathematica software.

Supplementary Note 1

Computational results: The simulated results show different power-dependent behaviors of the upconverting Tm^{3+} emission bands without and with consideration of Tm^{3+}-Tm^{3+} cross-relaxation (Supplementary Figure 10b). First, the presence of Tm^{3+}-Tm^{3+} cross-relaxation leads to the ease of observation excitation saturation (Supplementary Figure 11a and b). Furthermore, the presence of Tm^{3+}-Tm^{3+} cross-relaxation results in less difference in the power dependence of two- and three-photon upconversion emissions (Supplementary Figure 11b). These two points match well with the experimental power dependence of Tm^{3+} emission (Supplementary Figure 11c). These results suggest that the emission of Tm^{3+} at 345 nm is likely to be a result of a five-photon upconversion process other than a four-photon upconversion process because of the presence of Tm^{3+}-Tm^{3+} cross-relaxation. More importantly, the Mn^{2+} upconversion emission exhibits essentially the same power dependence to the five-photon upconversion of Tm^{3+} ions as their linear fitting curves at a low excitation power density region show a negligible difference in the slope (Supplementary Figure 11d). Taken together, these findings suggest that the upconversion emission of Mn^{2+} center at 550 nm is originated from a five-photon process, and the lower exponential power dependence is experimentally observed due to the occurrence of cross-relaxation between neighboring Tm^{3+} ions. Note that this argument is consistent with previous results observed in cubic-phased Mn^{2+}-doped $NaGdF_4$ nanoparticles.[4]

Supplementary References

(1) Mai, H. et al. High-quality sodium rare-earth fluoride nanocrystals: controlled synthesis and optical properties. *J. Am. Chem. Soc.* **128**, 6426–6436 (2006).

(2) Zeng, S. et al. Simultaneous realization of phase/size manipulation, upconversion luminescence enhancement, and blood vessel imaging in multifunctional nanoprobes through transition metal Mn^{2+} doping. *Adv. Funct. Mater.* **24**, 4051–4059 (2014).

(3) Wang, F., Deng, R. & Liu, X. Preparation of core-shell $NaGdF_4$ nanoparticles doped with luminescent lanthanide ions to be used as upconversion-based probes. *Nat. Protoc.* **9**, 1634–1644 (2014).

(4) Li, X. et al. Energy migration upconversion in manganese(ii)-doped nanoparticles. *Angew. Chem. Int. Ed.* **54**, 13312–13317 (2015).

(5) Bogdan, N., Vetrone, F., Ozin, G. A. & Capobianco, J. A. Synthesis of ligand-free colloidally stable water dispersible brightly luminescent lanthanide-doped upconverting nanoparticles. *Nano Lett.* **11**, 835–840 (2011).
