# Supplementary material for: Binary temporal upconversion codes of Mn2+-activated nanoparticles for multilevel anti-counterfeiting
Source: Nat Commun. 2017 Oct 12;8:899. doi: 10.1038/s41467-017-00916-7 (PMC5638907; doi:10.1038/s41467-017-00916-7)
Supplement: Supplementary file 2 — Description of Additional Supplementary Files [file 41467_2017_916_MOESM2_ESM.pdf]

Description of Additional Supplementary Files

File Name: Supplementary Movie 1
Description: Binary temporal upconversion codes can be visualized by scanning a nanoparticle-covered substrate with a 980 nm laser beam (64 W cm-2). The main spot emission is a result of mixture upconversion luminescence of lanthanides and manganese (II) ions, while the tail emission arises from the delayed upconversion emission of manganese (II) ions. The presence of the tail emission can largely enhance the complexity of anti-counterfeiting feature while maintaining the process of authentication at throughput rates.
